# Sexual behavior, risk perception, and HIV transmission can respond to HIV antiviral drugs and vaccines through multiple pathways

**DOI:** 10.1038/srep15411

**Published:** 2015-10-28

**Authors:** Stephen Tully, Monica Cojocaru, Chris T. Bauch

**Affiliations:** 1 Department of Mathematics and Statistics University of Guelph, 50 Stone Road East, Guelph, ON, N1G 2W1 Canada; 2 Department of Applied Mathematics University of Waterloo, 200 University Avenue West, Waterloo, ON, N2L 3G1 Canada

## Abstract

There has been growing use of highly active antiretroviral treatment (HAART) for HIV and significant progress in developing prophylactic HIV vaccines. The simplest theories of counterproductive behavioral responses to such interventions tend to focus on single feedback mechanisms: for instance, HAART optimism makes infection less scary and thus promotes risky sexual behavior. Here, we develop an agent based, age-structured model of HIV transmission, risk perception, and partner selection in a core group to explore behavioral responses to interventions. We find that interventions can activate not one, but several feedback mechanisms that could potentially influence decision-making and HIV prevalence. In the model, HAART increases the attractiveness of unprotected sex, but it also increases perceived risk of infection and, on longer timescales, causes demographic impacts that partially counteract HAART optimism. Both HAART and vaccination usually lead to lower rates of unprotected sex on the whole, but intervention effectiveness depends strongly on whether individuals over- or under-estimate intervention coverage. Age-specific effects cause sexual behavior and HIV prevalence to change in opposite ways in old and young age groups. For complex infections like HIV—where interventions influence transmission, demography, sexual behavior and risk perception—we conclude that evaluations of behavioral responses should consider multiple feedback mechanisms.

Over the past few decades, our growing understanding of factors contributing to HIV transmission has improved HIV control efforts. Partner selection and safe sex practices are now recognized as major determinants of HIV prevalence^1^,^2^ and have been analyzed through mathematical models focussing on behavioral aspects of HIV and other sexually infectious diseases^3^,^4^,^5^,^6^. This remains an important issue, particularly with data indicating that, despite greater public awareness, HIV incidence rates are once again increasing in men who have sex with men (MSM) in the United States^7^. Fortunately, treatment options such as Highly Active Antiretroviral Treatment (HAART), are becoming more widely available, and an effective prophylactic HIV vaccine is now a realistic goal^8^.

However, implementing treatment and prevention programs may have unintended consequences. Interventions like HAART that delay progression to AIDS may reduce the fear of living with HIV/AIDS (so-called ‘HAART optimism’). This in turn may cause individuals to choose riskier sexual behaviors, thereby increasing HIV transmission more than would have been the case if sexual behavior did not change due to HAART^9^,^10^. Alternatively, both prophylactic vaccines and HAART may reduce HIV prevalence, which in turn may also cause some individuals to become complacent about their risk of infection and thus increase their risky sexual behavior, thereby also increasing HIV transmission. Both types of responses are examples of ‘policy resistance’, where the population’s response to an intervention tends to undermine the intervention^11^. This could potentially lead to an increase in HIV prevalence, particularly in high risk groups such as MSM (men who have sex with men)^12^.

As such, it is important to assess a population’s perception of how the risks associated with contracting HIV might evolve due to introducing HAART or prophylactic vaccines, and the resulting changes in disease dynamics. Cross-sectional surveys can provide valuable information on a population’s perception and behavior at a specific time. However, it is difficult to extrapolate from surveys to determine how perception and behavior might evolve under different circumstances, such as when a new intervention is introduced to the population (indeed, it is only possible to gauge how individual’s think they would respond under different circumstances). In contrast, mathematical models can be used to extrapolate to different circumstances, but they also require making simplifying assumptions. Combining insights from both mathematical models and surveys may provide a clearer understanding of how policy resistance to HIV interventions can emerge. Using empirically informed mathematical models in particular may help us understand how HIV interventions change the interplay between individual risk perception, sexual behavior, and population disease dynamics.

HAART optimism is the focus of many surveys designed to measure how risk perception evolves after introducing HIV interventions. HAART optimism is defined as a consequence of HAART that may lead to a change in behavior, such as an increase in unprotected sex, or a decreased likelihood to remain on medication. A number of studies have explored the existence of HAART optimism, and the factors that may drive its emergence. In terms of treatment commitment, one such study comprising of a mass questionnaire found that 57% of optimists reported not always taking their HAART, compared to 29% of the individuals characterized as pessimistic^13^. The study authors concluded that treatment centres should more closely monitor patients’ prognostic beliefs.

Studies have come to varying conclusions regarding the impact of HAART optimism on sexual behavior. Some studies have found an increased level of risky behavior in HAART patients^9^,^10^,^14^. For example, Huebner *et al.* conducted a mass survey to better understand the relation between gay and bisexual men’s beliefs about HAART and sexual risk behavior. Using study participant data, Huebner found evidence of risk perception changing due to treatment. Men who believed that HAART decreased the risk of HIV transmission expressed reduced intentions to use condoms for anal sex and were also more likely to have engaged in unprotected anal intercourse with a casual partner. The behavioral surveys extend to the impact of potential HIV vaccines on sexual behavior. Lee *et al.* developed an HIV attitudes scale to predict vaccine acceptability. The survey randomly sampled individuals attending STD clinics and needle exchange sites. Risk compensation was found to be positively and significantly associated with vaccine acceptability, indicating individuals open to adopting the HIV vaccine perceived less of a need for protection through condom use^15^.

Other studies have found that HAART caused no change in risky behavior^16^, or decreased risky behavior^17^. For instance, Remien *et al.*’s cross-sectional study in MSM found no increased risk behavior associated with HIV positive individuals being on HAART^16^. Stephenson *et al.* conducted a cross-sectional study of HIV positive homosexual men, finding that men on HAART had fewer sexual partners, less unprotected anal intercourse and fewer acute sexually transmitted infections than men not on HAART. These cross-sectional studies studies illustrate the potential complexity of perceptual and behavioral responses to interventions. Differing study conclusions may reflect different study populations or study design. Moreover, because disease dynamics unfold over time, cross-sectional studies may fail to capture how disease dynamics may also influence risk perception and sexual behavior.

Epidemiological models such as disease transmission models or game theoretical models can be used to address how disease dynamics unfold over time and influence risk perception and behavior in a single population^3^,^4^,^5^,^6^,^18^,^19^,^20^. Previous disease transmission models have addressed the issue of how HAART optimism may occur and how it may influence disease dynamics and/or behavior. For example, Ramadanovic *et al.* focus on changing risk behaviors in the context of preventive HIV treatment^3^. The authors create a deterministic compartmental model that treats HIV risk behavior and infection as linked processes wherein the individual increases risk behavior as a precursor to contracting HIV. Ramadanovic *et al.* demonstrate that HIV incidence and prevalence only decline above threshold levels of HAART coverage, indicating a strong connection that is dependent on risk behavior parameter values. Results showed that expanding HAART coverage combined with interventions aimed to reduce risky behaviors would amplify the preventative impact and possibly eliminate the HIV epidemic. A model of HIV and vaccination by Smith? *et al.*, defines a fitness ratio as the average number of secondary HIV infections caused by an infected vaccinated individual, divided by the basic reproduction number^21^. By holding risk behavior constant, they were able to determine thresholds at which HIV prevalence would increase instead of decrease due to the vaccine, in the case that disease modifying vaccines provide only a low degree of protection against infection and/or generate high fitness ratios. Their findings report that the success of a vaccination campaign hinges upon the fitness ratio, the proportion of the population that were vaccinated, and the degree of change of risk behavior in unvaccinated infected individuals^21^.

Game theoretical and other behavioral modeling frameworks formalize how individuals make decisions in a strategic environment. Schroeder *et al.* used a signalling game to investigate sexually transmitted disease epidemics. They constructed simple signalling game models which demonstrate that uninfected individuals engage in high-risk sex with both uninfected and potentially infected partners, if the perceived prevalence of infection is sufficiently low^22^. Applying a decision-making model to infectious diseases, Auld investigated how a population’s choices and beliefs impact infectious diseases and the spread of HIV. Using a dynamical model, he showed that the success of an intervention is highly dependent on whether the population anticipates the presumed changes and adjusts their behavior. This demonstrated that a population’s behavior leading up to introduction of an intervention could impact its spread, and thus is important to consider^23^.

Much of the previous literature on HIV transmission and behavioral modelling focuses on population responses to interventions based on single mechanisms, such as exploitation of herd immunity. Here, our objective is to understand how populations can respond to the introduction of interventions through multiple mechanisms (pathways). We develop an agent-based model to explore the impact of coevolution of risk perception, sexual behavior, and HIV transmission dynamics in the context of prophylactic vaccines and HAART. Our model captures the interaction between the sexual partner selection process (as it depends upon time-evolving individual risk perception) and HIV disease dynamics, and how both respond to the introduction of HAART and prophylactic vaccines. Our objective is to explore potential mechanisms that may either lead to, or prevent, the development of policy resistance against those interventions. Each individual uses a utility function to decide whether to practice protected sex (PS), unprotected sex (US), or no sex (NS) with the other individual they are interacting with. (Utility is an abstract measure of preference that individuals can use to compare and rank different possible outcomes). This approach helps identify how decisions are formed, and how interactive decision-making between HIV+ and HIV− individuals helps determine spread of HIV. We introduce heterogeneity into the model in the form of age structure, allowing us to explore age-specific effects. With a more informed understanding of how risk perception evolves, and how it reacts to the introduction of HAART and prophylactic vaccines in a population, we can better understand the changes in behavior associated with new treatment options. We use the model to shed light on the complex nuances of HAART optimism and vaccine policy resistance.

## Methods

The model represents HIV transmission and partner selection dynamics in a core group of individuals with higher partnership turnover, such as a core group of MSM. This was motivated by studies such as Gomes do Espirito Santo *et al.*, that describe how the clients of male sex workers can act as a bridge for HIV transmission to the general population^24^. Sex workers and their clients are also the target of many prevention initiatives, which is why such groups were chosen as our main focus. The model is based on a previous agent-based partner selection model^25^. The previous model is a simplified representation of the co-evolution between risk perception and HIV prevalence, within a homogeneous population of MSM over a relatively short amount of time. Individuals are randomly paired and, while aware of their own status, are unaware of their partner’s status. The game they play represents a strategic interaction between the individuals, wherein utilities are weighed and an outcome, dependent on how an interaction has progressed, is chosen.

The previous model was modified to incorporate: (1) age structure, including age-specific sexual activity levels and mixing patterns, (2) vital dynamics, including recruitment, death and aging, (3) a modified representation of partner selection, (4) modifications to utility functions and risk perception dynamics to accommodate the presence of HAART and/or vaccine programs in the population, and (5) the impact of HAART and prophylactic vaccines on HIV infection probabilities and HIV natural history.

### Vital Dynamics and Partner selection

Every time-step represents one month, after which each individual’s age counter is advanced by one month. New individuals enter the sexually active population at age 15 years^26^. All individuals will exit the population through natural death at 79 years of age, or preemptively by death due to AIDS.

In each time step, there is a probability that an individual engages in an encounter that may lead to sex (i.e., plays the ‘risky sex game’). The probability of engaging is highest in 20-year-olds and declines with age (see Supplementary Table S1)^27^,^28^. If an individual engages, they engage with a randomly selected individual who is within 8 years of their own age^26^. There is a random chance that any given actor will be assigned to be “Actor 1” in an interaction. Actor 2 represents the randomly chosen individual fitting the age requirement, and must not be engaged in a current interaction. Actor 2 must interact with Actor 1, if Actor 1 decides to interact with Actor 2 (although they do not have to choose sex). Individuals decide by calculating utilities based on the value of sex (protected or unprotected), the risk of contracting HIV (which depends on behavior of the other individual, perceived , and perceived vaccine coverage), the health impact of contracting HIV, and how the impact might be modified by HAART (see following subsection on HAART). A detailed description of the interaction between individuals in an encounter appears in the Supplementary material.

### Risk perception dynamics

In order to model risk perception evolution, we define *b*_*t*_ to be an individual’s personal risk assessment at time *t*. Each actor has a specific *b*_*t*_ value. This value varies over the course of the simulation as actors update their *b*_*t*_-value based on their encounters. Each actor draws their initial *b*_*t*_-value *b*_0_ randomly from a normal distribution of mean *μ* = 0.05 and standard deviation *σ* = 0.1; sampled values less than 0 or greater than 1 are discarded. We chose a relatively small value for the initial *b*_*t*_-value because recruited individuals in the model are younger, and both the perceived prevalence of HIV in a population as well as the perceived risk of contacting HIV or other STIs are less within younger age groups^29^,^30^,^31^. During an interaction, an individual will either increase their risk assessment or decrease it depending on the offers they are receiving. If Actor 1 offers US, Actor 2 increases their *b*_*t*_-value according to


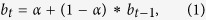


where *α* is called the “historical influence parameter”, since it measures how much of *b*_*t*_ is from past encounters versus the current offer. If Actor 1 offers PS, then Actor 2 decreases their *b*_*t*_-value according to


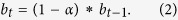


Risk perception changes by adjusting the assigned utilities as well as *b*_*t*_-value. Utilities are assigned to possible actions, and an Actor’s decision is dependent on the weight of the utility and their risk perception. The formulation for utilities and calculating the expected utility is similar to that described in the previous paper^25^ and is described further in the supplementary material. Prior to intervention, the utility for US for an HIV− individual engaging with an HIV+ individual is *U*_*US*_ = −50. However, with the introduction of HAART and its effects on lowering transmission and increasing life span, the *U*_*US*_ is increased to account for this. Depending on the HAART coverage (*ρ*), and *k* influencing the over or under-estimation of coverage in the population, the resulting *U*_*US*_ can become more appealing than NS.


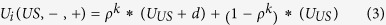


This expression captures how the utility for risky sex, in the presence of HAART, is a function of both utility for unprotected sex without the availability of HAART (*U*_*US*_) and the utility of unprotected sex if HAART is available to them (*U*_*US*_ + *d*), weighted according to the perceived availability of HAART in the population (*ρ* modified by misperception of true HAART coverage, *k*). By adjusting the utility, the choices made by individuals are altered and risky sex is perceived as more attractive under HAART.

The introduction of an HIV vaccine also changes the risky sex game. An HIV− individual that has been vaccinated and has acquired immunity will then alter how they update their *b*_*t*_-value as well as the *U*_*US*_ for engaging with an HIV+ individual, by changing this to be the same utility as interacting with an HIV− person. Once the Actor however realizes that they are no longer protected, they will revert back to an HIV− utility set (See Table 1). Once the vaccine is introduced, the method by which an individual updates their *b*_*t*_-value will change. Updating when someone offers US will now also be dependent on how the individual perceives the current vaccination coverage of the individual they are interacting with. When an individual is offered US, they will update their *b*_*t*_-value according to:


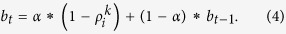


where we assume individuals know the age of the individual making the US offer, and *ρ*_*i*_ is the vaccine coverage in the age group *i* of the individual who is making the offer (see Table 1 for *ρ*_*i*_ values; *ρ*_*i*_ is set to zero 10 years after vaccination because we assume individuals know that the vaccine immunity wanes). We can adjust *k* to reflect an individual’s belief of the prevalence of the vaccine in the population. This parameter is included because of studies such as Herlitz *et al.* showing that individuals do not have perfect knowledge of HAART coverage in the population^32^. The adjustments to utilities, and changes to how individuals update their risk assessment, describe possible responses due to introducing HAART and/or prophylactic vaccines. Analysis of the individual risk perception time evolution aids in understanding risk perception dynamics.

### HIV transmission and natural history

A person infected with HIV passes through the acute, latent, pre-AIDS, and AIDS stages. The duration of each stage is a fixed time interval (Supplementary Table S2). Individuals die at the end of the AIDS stage (or the AIDS with treatment stage, see subsection on HAART below). The different stages of infection are illustrated by Supplementary Figure S1. During unprotected sex, an infected person infects a susceptible person with a probability dependent on their current infection stage, with the relative infectivity during the various stages being highly variable (Supplementary Table S3). However, individuals in the AIDS stage are assumed not to engage in sexual encounters at all, due to symptomaticity. In order to account for newly infected individuals unaware of their status who continue under the assumption they are HIV−, we introduced *ζ* = 0.1 (Table 2), meaning that an HIV infected individual has a 10% chance of being tested (and thereby discovering their true HIV status) per timestep (one month). We note that the proportion of individuals unaware of their HIV status varies significantly between populations, and in many populations this proportion is higher than what we have assumed here^33^.

### HAART

There is considerable debate as to when HAART should be initiated, since the benefits of early treatment can be counteracted by antiviral drug toxicity^34^. Here, we assume that at the end of the latent stage, an individual enters HAART with probability *ρ*, where *ρ* represents treatment coverage. HAART lengthens life, facilitates relative reduction in transmission, and improves the utility that an HIV− individual associates with unprotected sex, by an additive factor *d* (see Supplementary material). Since HAART availability improves the *U*_*US*_, it affects whether individuals offer, or accept, unprotected sex, and hence it may influence the perceived risk parameter, *b*.

The proportion of people living with AIDS who were receiving HAART increased from 74% in 2005 to 90% in 2008 in San Francisco^35^. In lower-income countries, the coverage is much lower but is rapidly increasing^36^. Here we consider values of *ρ* with a baseline of 75% and simulate coverage from 0 to 100%. In simulations, we allow the population to come to an epidemiological and demographic equilibrium before introducing HAART into the population.

### Prophylactic HIV Vaccine

Drawbacks of HAART include drug resistance, drug toxicity, and the need to know HIV status and CD4 count before offering intervention. As a result, more than 30 HIV vaccines are being tested in human clinical trials worldwide^8^, including at least one based on a genetically modified killed whole virus^37^. A prophylactic vaccine would mean that a certain percentage of vaccinated HIV− individuals are protected from infection, even if they engage in unprotected sex. As a result, prophylactic vaccines could impact how individuals update their risk perception based on their partner actions, and knowledge of vaccine coverage in the population.

A randomly chosen proportion *ρ* of 15-year-olds entering the sexually active population receive the vaccine, which has all-or-none efficacy 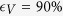. Similar to the practice with hepatitis B vaccine in some jurisdictions, we assume that individuals are tested immediately after vaccination, and thus are aware if the vaccine worked for them. Individuals who were not vaccinated, or in whom the vaccine was ineffective, do not change their utilities. However, those who are efficaciously protected increase their utility for unprotected sex, since they cannot be infected (see Supplementary Material for details).

Vaccine scheduling recommendations depend on various factors, including epidemiological, social, and health economic factors. Our assumption for HIV vaccine program design was based on experience with the HPV vaccine, which was first made available in 11–15 year olds^38^. The reason for this is that the HPV vaccine is prophylactic (similarly to our assumed HIV vaccine) and thus, by definition, only protects susceptible persons, not infected persons. Hence, it is more efficient to vaccinate age groups where the proportion of susceptible individuals is highest, so vaccination is not wasted on individuals who are already infected. The model does not however, incorporate catch-up programs which would likely be rolled out in older age classes.

The duration of protection for an efficaciously vaccinated individual is taken from a normal distribution with a mean 10 years and a standard deviation of 3 years. Vaccines are tested 5 and 10 years after being vaccinated and if the test indicates that their vaccine protection has ceased, the individual will change their utility back to that of an HIV− individual, otherwise they will continue with the utility of a vaccine-protected HIV− individual. At 15 years post-vaccination, individuals assume they are no longer protected and revert to the utility function of an unprotected HIV− individual. As no HIV vaccine currently exists, our testing assumption is based on practice associated with the Hepatitis B vaccine, which also protects against a dangerous sexually transmitted infection, and for which individual immunity after vaccination is tested in some jurisdictions^39^.

## Results

The partner selection model is based on a previous agent-based model used to explore the coevolution of risk perception, partner selection, and HIV transmission^25^. In the present model, individuals in an age-structured core group population (1) engage with others, (2) decide whether to have protected or unprotected sex based on their own status, the perceived probability that the other person is HIV+, and utilities, as modified by the presence of HAART and vaccines, and (3) may transmit the infection if they have unprotected sex. The model is outlined in the Methods Section.

We investigate changes in the population caused by HAART or prophylactic vaccines by comparing the dynamics with and without the interventions.The simulations are run to equilibrium before an intervention is introduced. Because the model is necessarily complicated on account of exploring multiple potential feedbacks on perception and behavior, we focus on the impact of these interventions separately and do not investigate scenarios where both are applied, leaving this to future work.

We explore how these changes depend on model parameters governing vaccine and treatment coverage, vaccine efficacy, duration of protection, risk perception parameters, and transmission rates. The interventions are implemented at year 50 of the simulation in all figures, by which time the population has reached equilibrium. We also vary the utility associated with a sexual act, the value of which contributes to the decision making process. Model outcomes include population sizes, HIV prevalence, the perceived probability that the other person is HIV+ (which we hereafter refer to simply as “perceived infection risk”), and per capita number of unprotected sex acts (US) (i.e. US acts per HIV− individual, providing a gauge of risky behavior before and after interventions), broken down by age and HIV status. Baseline parameter values and utilities appear in Table 2 and Table 3 respectively. We use these parameter values in all simulations except when stated otherwise.

### Baseline scenario: no HAART or vaccines

**Figure 1:**
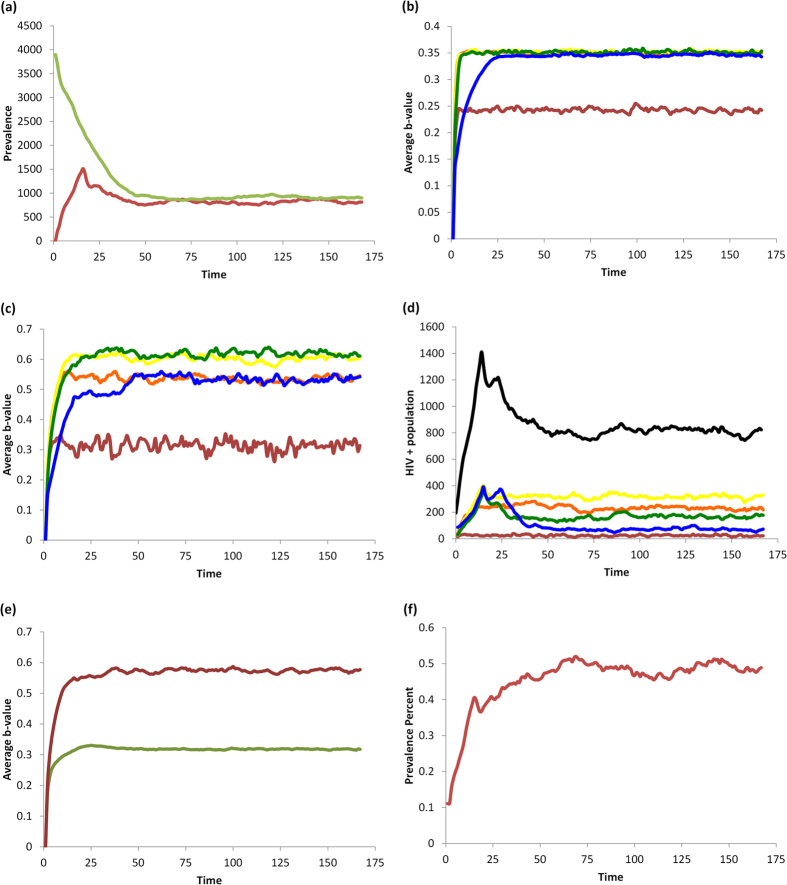
Baseline scenario with no interventions. (**a**) number of HIV—(green) and HIV+ (red) individuals in the population; (**b**) average *b*_*t*_-value for HIV—individuals by age, 15-20-year-olds (red), 20–30 (orange), 30–40 (yellow), 40–50 (green), and 50+ year-olds (blue); (**c**) average *b*_*t*_-values for HIV+ individuals by same age groups; (**d**) number of HIV+ individuals by the same age groups, and with black representing the total number of HIV+ individuals; (**e**) total average *b*_*t*_-value for HIV+ (red) and HIV—(green) populations; (**f**) HIV prevalence (percentage of the population currently infected).

**Figure 2:**
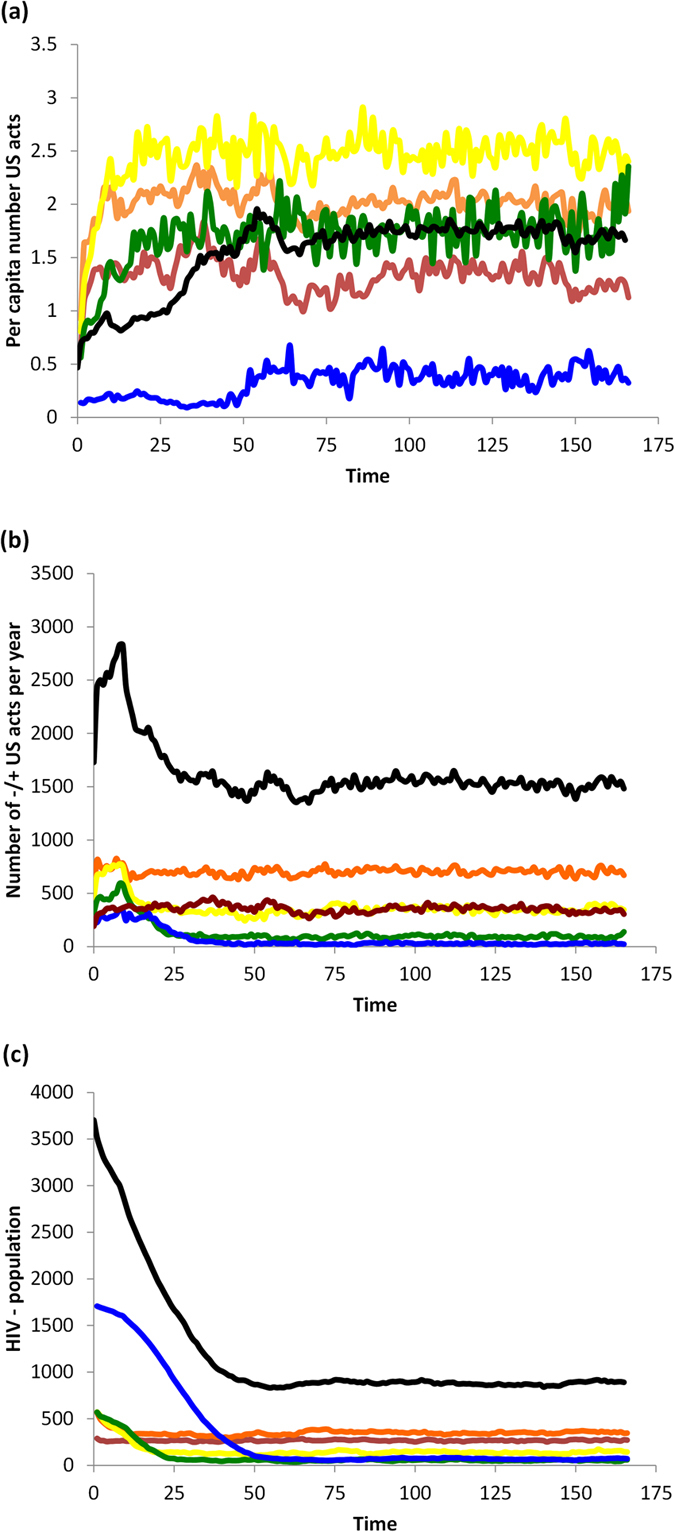
Baseline scenario with no interventions. (**a**) total number of −/+ US acts (number of unprotected sex acts between HIV− and HIV+ pairs) per year divided by number of HIV—individuals in that age cohort at the end of the year, for age groups 15–20-year-olds (red), 20–30 (orange), 30–40 (yellow), 40–50 (green), and 50+ year-olds (blue), and cumulative number of individuals across all age groups (black); (**b**) total number of −/+ US acts per year, for same age groups; (**c**) number of HIV—individuals in each age cohort at the end of the year, for same age groups.

In the absence of interventions such as HAART or vaccines, the population converges to equilibrium levels of population size, HIV prevalence, and perceived infection risk (*b*) (Figs 1 and 2). HIV prevalence is highest in 20–29 and 30–39 year olds. The average perceived infection risk *b*_*t*_ is similar to the HIV prevalence, although HIV− individuals tend to underestimate HIV infection risk and HIV+ individuals tend to slightly overestimate it. The perceived infection risk tends to be lowest in 15–19 year olds.

As expected, increasing the transmission rate *τ* increases HIV prevalence, as well as the average *b*_*t*_-value (perceived infection risk) (Supplementary Figure S2). Increasing the utility for protected sex (*U*_*PS*_) decreases both the HIV prevalence and the average perceived infection risk (Supplementary Figure S3). This occurs because having a more attractive utility for PS reduces the spread of HIV and hence the perceived risk of contracting HIV.

### Impact of HAART on risk perception, sexual behavior, and HIV prevalence

**Figure 3:**
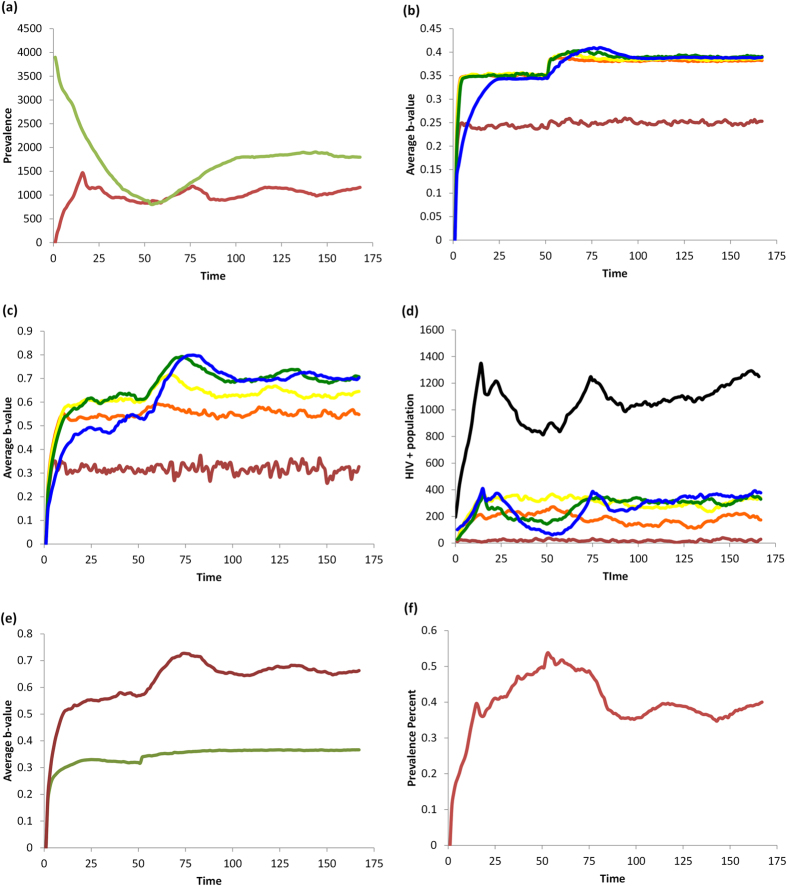
Baseline scenario for HAART intervention. (**a**) number of HIV− (green) and HIV+ (red) in the population; (**b**) average *b*_*t*_-value for HIV− individuals by age, 15–20-year-olds (red), 20–30 (orange), 30–40 (yellow), 40–50 (green), and 50+ year-olds (blue); (**c**) average *b*_*t*_-values for HIV+ individuals by same age groups; (**d**) number of HIV+ individuals by the same age groups, and with black representing the total number of HIV+ individuals; (**e**) total average *b*_*t*_-value for HIV+ (red) and HIV− (green) populations; (**f**) HIV prevalence (percentage of population currently infected).

Parameters for the baseline HAART scenario appear in Table 1. HAART decreases the transmission rate and thus reduces the incidence of new HIV cases. However it also increases the lifespan of HIV+ individuals, such that the net impact of HAART is a small net increase in the number of HIV+ individuals in the population (Fig. 3a). Significant reductions in the number of HIV+ individuals in the 15–39 year old age range due to reduced incidence are offset by significant increases in the number of HIV+ individuals in the 40+ age range due to longer lifespans and delayed age at infection (Fig. 3d). As a result of reduced transmission rates, the number of HIV− individuals grows considerably, causing a decrease in overall HIV prevalence (Fig. 3a,f).

**Figure 4:**
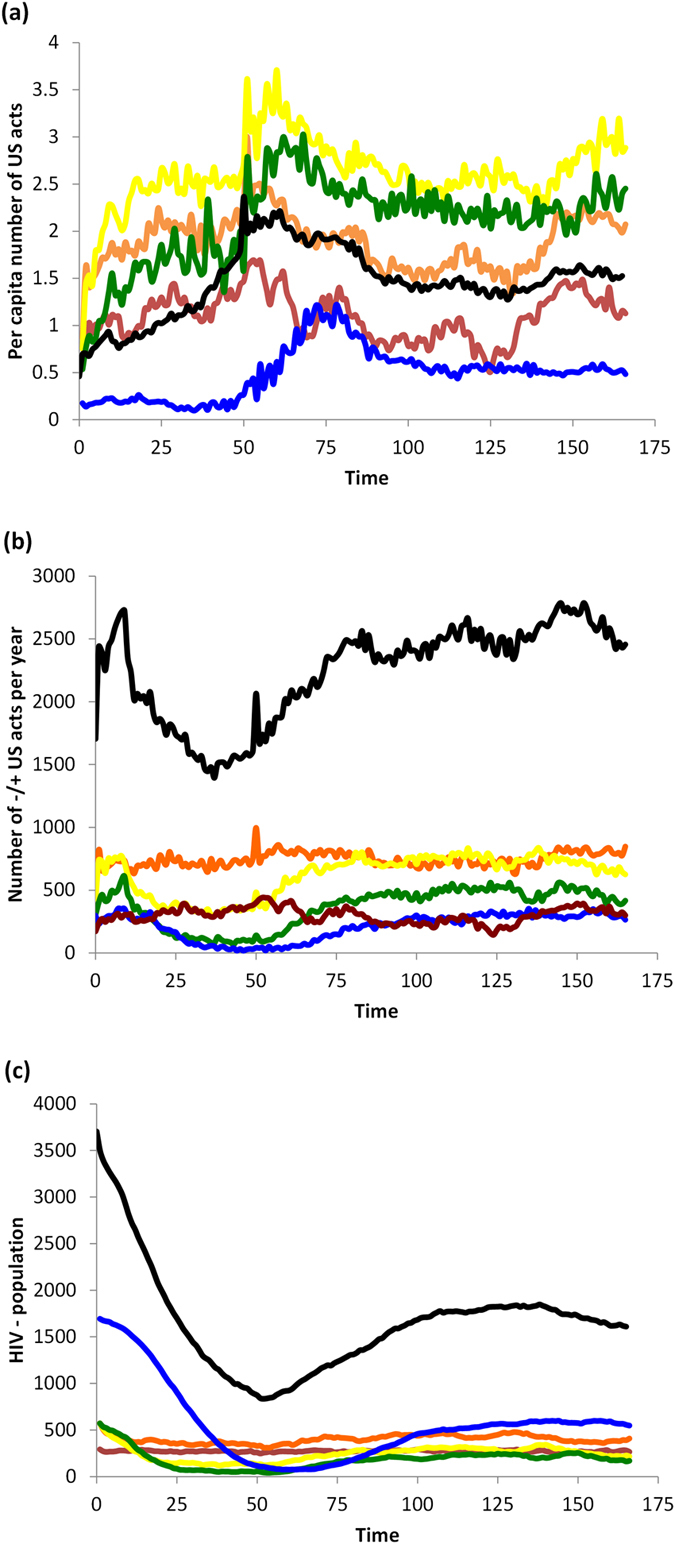
Baseline scenario for HAART intervention. (**a**) total number of −/+ US acts (number of unprotected sex acts between HIV− and HIV+ pairs) per year divided by number of HIV—individuals in that age cohort at the end of the year, for age groups 15–20-year-olds (red), 20–30 (orange), 30–40 (yellow), 40–50 (green), and 50+ year-olds (blue), and cumulative number of individuals across all age groups (black); (**b**) total number of −/+ US acts per year, for same age groups; (**c**) number of HIV—individuals in each age cohort at the end of the year, for same age groups.

In the short-term, HAART optimism causes a transient spike in the per capita rate of unprotected sex (US) between HIV+ and HIV− individuals in all age groups (Fig. 4a versus Fig. 2a). There is a corresponding increase in the total number of US acts, driven primarily by older age groups where the HIV+ population is expanding (Fig. 4b versus Fig. 2b). HAART makes unprotected sex more attractive to HIV− individuals by increasing the utility of US through the parameter *d*, which in turn increases the number of US offers.

However, two other pathways partially counteract the effect of HAART optimism. On account of Equation 4, the increase in the number of US offers also increases the average value of *b*_*t*_ representing the perceived infection risk, across most age groups, and for both HIV+ and HIV− individuals (Fig. 3b,c,e versus Fig. 1). The increase in perceived infection risk has a protective effect on the population, by making HIV− individuals more likely to believe the other actor is HIV+ (since more US offers are being made). Additionally, on account of fewer new infections, a gradual but significant expansion of the HIV− population occurs over time (Fig. 4(c) versus Fig. 2(c)). This growth in the HIV− population exceeds the expansion of the HIV+ population (Fig. 4(d) versus Fig. 2(d)), resulting in a net reduction in HIV prevalence. This means that any given encounter is more likely to be with an HIV− person than an HIV+ person. As a result, for younger age classes, and across all age classes on average, the per capita rate of US between HIV+ and HIV− actors falls to an equilibrium value that is below the baseline scenario with no HAART, although in older age groups it falls to a level that remains above the baseline scenario with no HAART. Hence, in the long run, HAART results in less US being practiced by HIV− individuals on average, but not for older individuals. Both protective pathways tend to decrease the per capita number of unprotected sex acts between HIV+ and HIV− individuals in the long term, so that the transient spike in the per capita rate of US between HIV+ and HIV− actors due to HAART optimism is followed by a gradual decrease.

In summary, HAART causes HAART optimism in the short term along with a transient spike in unprotected sex acts between HIV+ and HIV− individuals. However, in the longer term, it activates other pathways that provide protective effects by modifying risk perception and population composition. The effect of HAART on multiple pathways—some of which are protective and some of which are not—illustrates the complex nature of interactions between interventions, individual risk perception and behavior, and population dynamics. This suggests one reason why different surveys of HAART effects conducted at different times in different populations might provide different results^9^,^10^,^16^,^17^. These results also illustrate how the impact of HAART can evolve over time, and change from net negative influences to net positive influences as the disease dynamics unfold.

### Univariate sensitivity analysis for the impact of HAART on the utility for unprotected sex (*d*)

At baseline parameter values, the benefits of HAART in reducing transmission and increasing the perceived infection risk are strong enough to counteract how HAART optimism makes US more attractive, resulting in a net reduction in HIV prevalence and incidence. HAART optimism operates through the model parameter *d*, which increases the utility of unprotected sex (Equation 3).

As the value of *d* is increased above the baseline value *d* = 20, US becomes increasingly attractive, which increases actual post-treatment HIV prevalence as well as the average perceived infection risk (Supplementary Figure S4). For *d* > 60, the post-treatment HIV prevalence exceeds the pre-treatment HIV prevalence: for these parameter values, the effects of HAART optimism cause a net increase in HIV in the population. At *d* = 60 for an HIV− actor, US with an HIV+ actor still provides a lower utility than PS. Hence, sufficiently strong HAART optimism could actually cause a net increase in HIV prevalence.

Decreasing *d* has the opposite effect, making US under HAART less attractive to HIV− individuals and thus decreasing HIV prevalence (with an accompanying decrease in perceived infection risk) (Supplementary Figures S5, and S6).

### Univariate sensitivity analysis for amount of over- or under-estimation of population HAART coverage (*k*)

The parameter *k* controls whether individuals over-estimate or under-estimate the amount of HAART coverage in the population. When *k* > 1 (respectively *k* *<* 1), individuals underestimate (respectively, over-estimate) the amount of coverage. This has implications for whether they think the other actor is HIV+ or not. If individuals underestimate HAART coverage (*k* > 1), then offers of US are more likely to be interpreted as meaning the other actor is HIV+, rather than being an HIV− person exhibiting HAART optimism, meaning their perceived prevalence of HIV will increase. Hence, they opt for protected sex, causing a decrease in HIV prevalence. This is indeed what is observed in simulations: as *k* increases, HIV prevalence decrease (Supplementary Figure S7). However, the average *b*_*t*_-value also decreases, since a decrease in HIV prevalence also causes a decrease in US offers. Hence, the decrease in US offers is offset by an increase in the likelihood that they are interpreted as evidence of HIV+ status, if individuals under-estimate HAART coverage in the population. The opposite happens for *k* < 1.

### Univariate sensitivity analysis for coverage (*ρ*), the transmission rate (*τ*) and the utility for protected sex (*U*_*PS*_)

Increasing the population coverage of HAART (*ρ*) decreases HIV prevalence, while the average perceived infection risk, *b*_*t*_, increases slightly, at baseline parameter values (Supplementary Figure S8). Increasing the transmission rate (*τ*) increases the HIV prevalence, and the perceived infection risk among HIV+ individuals, but has no effect on the perceived infection risk among HIV− individuals (Supplementary Figure S9). This occurs due to the structure of the game and the utilities assigned to the available actions (See Supplementary Section Understanding the evolution of *b*_*t*_-values). Finally, increasing the utility associated with protected sex (*U*_*PS*_) makes it more attractive, and decreases HIV prevalence as well as perceived infection risk among both HIV+ and HIV− individuals (Supplementary Figure S10).

### Impact of prophylactic vaccination

The baseline parameters for prophylactic vaccination appear in Table 1. Whereas HAART is provided to HIV+ individuals, prophylactic vaccination is provided to HIV− individuals. We assume an efficaciously vaccinated HIV− individual derives the same utility for US as an HIV+ individual, since they cannot acquire the infection while protected, which makes US offers attractive to vaccinated individuals.

**Figure 5:**
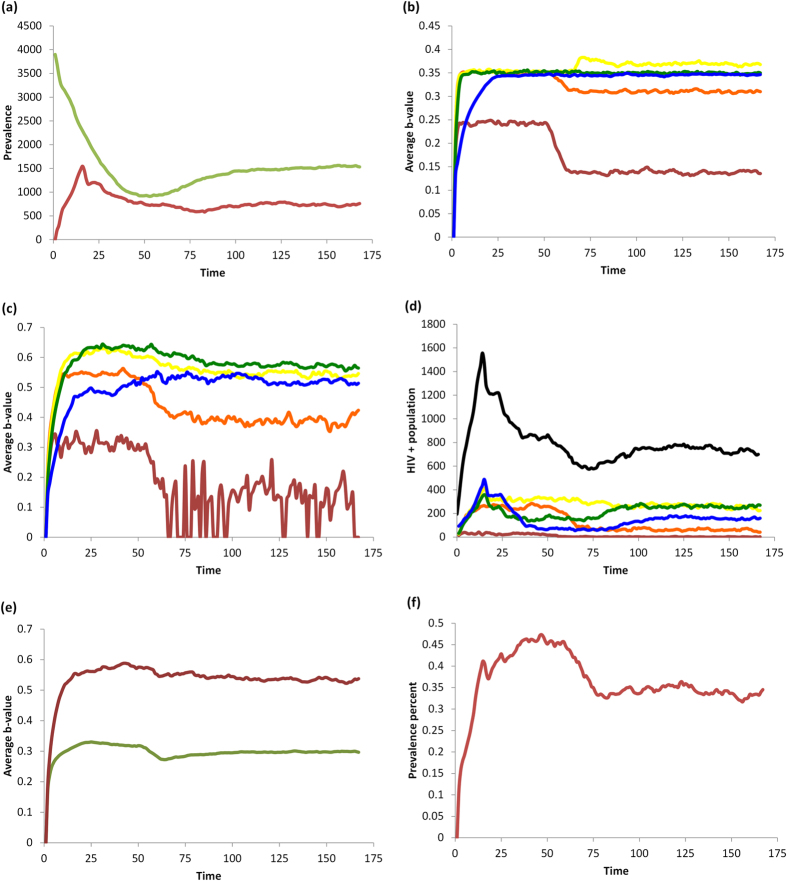
Baseline scenario for vaccine intervention. (**a**) number of HIV− (green) and HIV+ (red) in the population; (**b**) average *b*_*t*_-value for HIV− individuals by age, 15–20-year-olds (red), 20–30 (orange), 30–40 (yellow), 40–50 (green), and 50+ year-olds (blue); (**c**) average *b*_*t*_-values for HIV+ individuals by same age groups; (**d**) number of HIV+ individuals by the same age groups, and with black representing the total number of HIV+ individuals; (**e**) total average *b*_*t*_-value for HIV+ (red) and HIV− (green) populations; (**f**) HIV prevalence (percentage of population currently infected).

Implementation of a vaccination program at year 50 covering 75% of entering 15-year-olds, and providing protection for approximately 10 years, causes a rapid decrease in infected 15-29-year-olds (Fig. 5d). The intervention causes a particularly rapid reduction in HIV prevalence in the 15–20 year-old age group (Fig. 5d). Because HIV becomes so rare in this group, its dynamics are highly stochastic and caused corresponding changes in the offers of US versus PS, which in turn causes great variability in the value of *b*_*t*_ over time in HIV+ individuals in this age group (Fig. 5c). A delayed and more moderate decrease in HIV prevalence occurs in infected 30–39 year-olds, but the number of infected individuals actually increases in 40+ year-olds (Fig. 5d). This occurs because individuals who normally would have been infected when young, are no longer becoming infected due to the vaccine, but instead they become infected when they are older and vaccine protection has worn off. As a result, the total number of infected individuals remains relatively constant after vaccination is introduced, although the number of HIV− individuals increases, which reduces the HIV prevalence (Fig. 5a,f).

Individuals have some awareness of the age at which the vaccine is administered, the vaccine coverage, and the duration of protection, although they may overestimate or underestimate the actual vaccine coverage according to the parameter *k*. The vaccine program decreases the perceived prevalence of HIV among HIV− individuals under 29 years of age, causes a slight increase in perceived prevalence in 30–39 year olds, but it has no effect in 40+ year olds (Fig. 5b). The slight increase in 30–39 year olds occurs because some of those individuals mix with individuals in younger age classes who are still protected by the vaccine and hence have a preference for US.

Results are similar among HIV+ individuals, except the perceived prevalence decreases slightly in 30+ year olds (Fig. 5c). The decrease in perceived prevalence in younger age categories occurs due to awareness of the vaccine program, meaning that individuals are more likely to interpret an offer of US as evidence of vaccinated status. Hence, the decline in perceived prevalence tracks the decline in actual prevalence, even though US is still offered and practiced.

**Figure 6:**
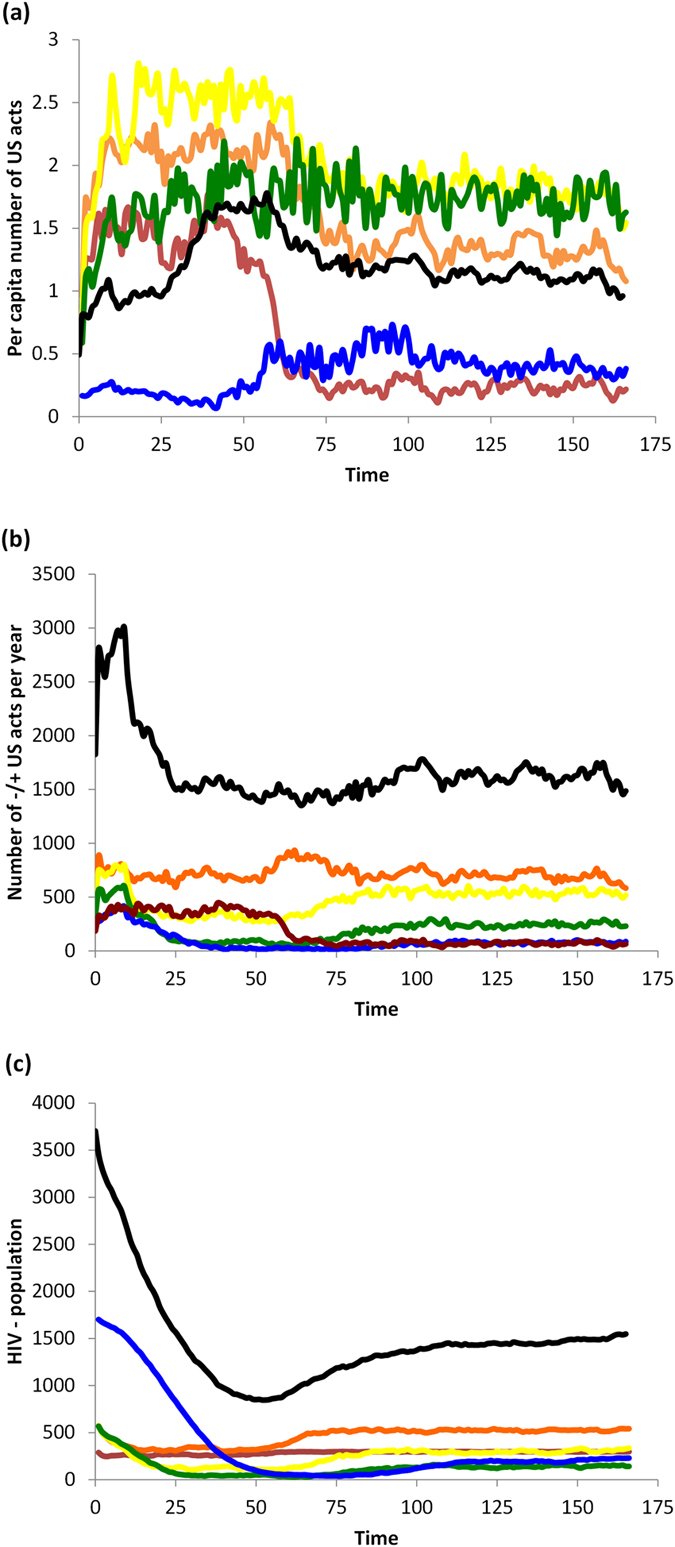
Baseline scenario for vaccine intervention. (**a**) total number of −/+ US acts (number of unprotected sex acts between HIV− and HIV+ pairs) per year divided by number of HIV—individuals in that age cohort at the end of the year, for age groups 15–20-year-olds (red), 20–30 (orange), 30–40 (yellow), 40–50 (green), and 50+ year-olds (blue), and cumulative number of individuals across all age groups (black); (**b**) total number of −/+ US acts per year, for same age groups; (**c**) number of HIV—individuals in each age cohort at the end of the year, for same age groups.

After the vaccine program is implemented, the rate of US acts between HIV+ and HIV− individuals per HIV− individual decreases in individuals below 39 years of age; remains constant in individuals 40–49 years old; and increases significantly in individuals 50+ (Fig. 6a). The decline in younger age categories reflects the significantly diminished number of HIV+ individuals in those age categories: there are simply not enough HIV+ individuals for per capita rates of US between HIV+ and HIV− actors to increase. In older age categories, the increase in per capita unprotected sex by HIV− individuals is caused by the increased population size of HIV+ individuals, due to the waning of protection from the vaccine.

Because (1) perceived infection risk tracks HIV prevalence (Fig. 5b,c) and (2) the per capita rates of unprotected sex are driven by changes in the numbers of HIV+ individuals in the age cohorts rather than behavioral changes stemming from altered utility functions *per se*, the negative feedbacks stimulated by prophylactic vaccination are limited. Although vaccine-protected individuals practice more US than without the vaccine, this does not translate into higher HIV prevalence. And, because vaccinated individuals are always HIV−, the direct impact on the other actor is minimal.

However, these results suggest that a booster program may be beneficial in older age categories, if vaccine protection wanes rapidly enough. Moreover, we did not explore the possibility of vaccine exemption due to vaccine-generated herd immunity, which could reduce vaccine coverage below socially optimal levels. The model also assumes individuals know their immuno-protection status, and that their vaccine protection can wane over time, which may not occur universally in real populations.

### Univariate sensitivity analysis for the over- or under-estimation of population vaccine coverage (*k*)

For higher values of *k*, such that individuals under-estimate population vaccine coverage, the perceived infection risk increases since individuals interpret US offers as evidence of HIV+ status. This causes HIV prevalence to decrease significantly compared to baseline, since PS becomes more attractive than US under such circumstances (Supplementary Figure S11). On the other hand, lower values of *k* mean individuals over-estimate population vaccine coverage, such that perceived infection risk decreases instead of increasing, and HIV prevalence declines only slightly compared to pre-vaccine prevalence (Supplementary Figure S12). Hence, underestimation of population vaccine coverage can significantly reduce the effectiveness of prophylactic HIV vaccination programs, due to the tendency to accept US more often. These observations are borne out across a range of value for *k* (Supplementary Figure S13). Also, changes in *k* have a greater effect for prophylactic vaccination than for HAART (Supplementary Figure S7).

### Univariate sensitivity analysis for vaccine coverage (*ρ*), vaccine efficacy ( 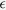 ), transmission rate (*τ*) and utility for protected sex (*U*_*PS*_)

Increasing the vaccine coverage *ρ* or the utility for protected sex *U*_*PS*_ decreases both actual and perceived infection risk, as expected (Supplementary Figures S14, S15). In contrast, increasing the vaccine efficacy 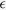 decreases the HIV prevalence but slightly increases the perceived infection risk (Supplementary Figure S16). This occurs because a higher vaccine efficacy protects more vaccinated individuals and thus leads to more US offers, however, individuals do not take vaccine efficacy into account in their risk perception, only vaccine coverage. Increasing the transmission rate *τ* increases the HIV prevalence (Supplementary Figure S17) similar to the observations found in the no intervention scenario.

## Discussion

This model illustrates how interventions, such as HAART and prophylactic HIV vaccines, can influence individual risk perception and behavior through multiple pathways. These interventions may result directly in HAART or vaccine optimism, since they may alter the utilities or perceived risks for an HIV− person interacting with someone who is offering unprotected sex. However, they also confer protective effects through other pathways. The first pathway is that optimism itself may increase the offers of unprotected sex, which can increase the perceived infection risk in the population and make some HIV− individuals more cautious than they would otherwise be. A second pathway occurs through disease dynamics: interventions reduce HIV transmission and prevalence, which increases the proportion of HIV− individuals in the population. This, in itself, protects HIV− individuals since any given sexual encounter is more likely to be with another HIV− individual, and hence the number of unprotected sex acts with HIV+ actors per HIV− individual decreases. At baseline parameter values, the decrease in per capita unprotected sex acts due to a changing composition of the population is a net decrease, i.e. the effects of changing population composition are outweighed by any increase due to optimism. We note that this is not a simple outcome of herd immunity, since individuals can choose protected sex or unprotected sex.

As a result of these other feedback loops, both HIV prevalence and the rate of unprotected sex acts with HIV+ actors, per HIV− individual decrease under both HAART and prophylactic vaccines, despite the effects of HAART and vaccine optimism. However, the decline is not as significant as it would have been if compensatory behavior did not emerge after the interventions. At other parameter values, HIV prevalence can increase as a result of the interventions. For example, we observed that when the effect of HAART optimism is sufficiently high, making the utility of unprotected sex sufficiently attractive, then HIV prevalence could actually rise under HAART (Supplementary Figure S4). Also, for both HAART and the vaccine, if individuals over-estimate the population coverage of the intervention, they will tend to interpret unprotected sex offers as evidence of HAART optimism or vaccine protection, rather than HIV+ status, which can have damaging consequences (Supplementary Figures S7, S13). Finally, if vaccine protection wanes after 10 years, then the burden of HIV incidence may simply be shifted to older age groups, which would necessitate booster programs.

This model makes several assumptions due to need for simplification or lack of data. Parameter values concerning prophylactic vaccines are based on preliminary assumptions since the effectiveness and duration of protection of such a vaccine were not known at the time of publication. In addition, the game theoretical model is a simplified representation of how individuals weigh their preferences; additional factors may influence the decision making process in real populations. The model does not include memory of past sexual encounters with specific individuals, or sexual network structure. Although the model includes age structure, there may be other heterogeneities that could influence results such as variable sexual activity levels, or social and cultural group identification. The model assumes that individuals who are offered unprotected sex increase their estimate of HIV prevalence in the population. This was motivated by studies that address risk factors and psychological determinants of behavior in HIV+ individuals^40^, showing that engaging in US is more frequent in HIV+ individuals. As such, since this is a relatively homogeneous and closed group with distinct norms and practices, we assume that individuals offered unprotected sex assume the other individual has a higher chance of being HIV+. However, in other populations, the opposite might be true.

The relative ranking of utilities for different choices is based on studies showing preferences for unprotected sex amongst highly active individuals. Although relative rankings are easier to establish, it is difficult to quantify the precise values of these utilities, which is why we varied the value of *U*_*PS*_ in the univariate sensitivity analysis. We found that increasing or decreasing this parameter influences prevalence and risk assessment as we might expect, but our primary qualitative conclusion that multiple pathways can determine behavior and prevalence remains unchanged under this sensitivity analysis. Furthermore, exploring the impact of homogeneously mixing core group dynamics on stable, concurrent partnerships with individuals outside the core group of interest (e.g. long-term partners) may also be relevant^41^. Future work could improve the realism of the model with respect to decision making processes and population structure (for instance, by allowing behavior and risk perception to be more heterogeneous in both HIV+ and HIV− groups); incorporate age-specific mortality rates; or attempt to replicate specific empirical findings concerning HAART. Future work could also better assess how prophylactic HIV vaccines could best be used, including optimal design of booster and catch-up programs.

We conclude that the behavioral pathways that are activated by introducing interventions are more complicated and numerous than the simple picture of potentially problematic or counter-productive behavioral responses usually suggests, and the pathways could be beneficial as well as harmful. Evaluations of the impact of HAART and potential prophylactic vaccines on risk perception and behavior should adopt a whole-population viewpoint regarding the effects of the intervention. This includes considering feedbacks that the interventions may introduce by altering dynamics of interaction between actors and their risk perception, population demographics (HIV prevalence), how these factors interact, and how they vary over time and across different age groups.

## Additional Information

**How to cite this article**: Tully, S. *et al.* Sexual behaviour, risk perception, and HIV transmission can respond to HIV antiviral drugs and vaccines through multiple pathways. *Sci. Rep.* **5**, 15411; doi: 10.1038/srep15411 (2015).

## Supplementary Material

Supplementary Materials

## Figures and Tables

**Table 1:** Parameter definitions and parameter values corresponding to HAART and vaccination scenarios.

Term	Definition	Value
*ρ*_*i*_	Treatment coverage (HAART or Vaccine). HAART coverage is independent from age group. Vaccine coverage is is implemented at age 15, with the age *i* impacting Equation 4	0.75 (*i* = 15–25), 0 (*i* = 26+)
d	adjustment to *U*_*US*_ due to HAART	20
k	parameter determining over- or under-confidence in coverage	1
*l*_*V*_	duration of vaccine protection	10 years (normal dist. 10, 3)
*I*_*t*_	times at which individuals are tested for remaining protection, post-vaccination	5, 10, and 15 years
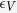	vaccine efficacy	0.9
*U*_*US*_(−, +)	utility for a HIV− individual to have US with a HIV+ individual, in absence of protection from the vaccine	−50
*U*_*US*_(−, +)(*V*)	utility for a HIV− individual to have US with a HIV+ individual, while assuming protection from vaccine.	100

**Table 2:** Parameter definitions and parameter values for baseline scenario.

Term	Definition	Baseline value
*b*_0_	Initial *b*_*t*_-value: initial value of *b*_*t*_ sampled from a beta distribution with mean 0.05 and a standard deviation of 0.1.	0.05
*α*	Historical influence parameter: measures how much of the *b*_*t*_-value will be based on past experience.	0.015
*τ*	Baseline transmission probability: probability of HIV transmission from an HIV+ actor to an HIV− actor through US. This value is modified according to stage of infection (see Supplementary Table S3)	0.02^42^
*U*_*PS*_	Protected sex utility.	50
*ζ*	Probability an individual who identifies as HIV− will seek testing to determine if they are infected.	0.1 per timestep

**Table 3:** Utilities for Actor 1, given status of Actor 2, in absence of interventions.

Actor 1 Status	Actor 2 Status	Utility for US	Utility for PS	Utility for NS	Preferences of Actor 1
HIV+	HIV+	100	50	0	*US* > *PS* > *NS*
HIV+	HIV−	100	50	0	*US* > *PS* > *NS*
HIV−	HIV+	−50	50	0	*PS* > *NS* > *US*
HIV−	HIV−	100	50	0	*US* > *PS* > *NS*

This outlines the preferences for different sexual acts given an individual’s status.
