# Supplementary material for: Sexual behavior, risk perception, and HIV transmission can respond to HIV antiviral drugs and vaccines through multiple pathways
Source: Sci Rep. 2015 Oct 28;5:15411. doi: 10.1038/srep15411 (PMC4623666; doi:10.1038/srep15411)
Supplement: Supplementary Materials [file srep15411-s1.pdf]

Sexual behaviour, risk perception, and HIV transmission can respond to HIV antiviral drugs and vaccines through multiple pathways

Stephen Tully, Monica Cojocaru, Chris T. Bauch

Supplementary Material

Utilities assigned in the risky sex game

The utility of an actor describes how an individual ranks their preferences. The formulation for utilities and calculating the expected utility is similar to that described in a previous paper [25]. The utility value $U_{k,i}(s_i, s_j)$ for actor i (1 or 2) of outcome k, unprotected sex, protected sex, and no sex, (k=US, PS, NS) and the HIV status of actor i is s_i (HIV+ or -) appear in Table 3. The numerical value for each utility is reflective of preference for each act. Dilley *et al*, found condom use to reduce pleasure and intimacy amongst men. For this reason, we rank US higher than PS, which in turn is higher than NS, except for the utility of an HIV- individual engaging with individual they believe to be HIV+ [43]. Moreover, many HIV+ men in some populations appear to be motivated to believe their partner is also HIV+ [44,45]. Similarly a study by Wolitski *et al*, of a group of HIV+ men found they often assumed an HIV- partner would make their status known before sex, and if they didn't, then they are likely HIV+ as well [46]. This is why the utility is assumed to be the same for an HIV+ individual engaging in US with either a HIV+ or HIV- partner. These values are used in calculating the expected utility (See Equation 5) which depends on b_t, an individual's *a priori* belief at time t of the probability that the person they are engaging with is HIV+ (we equate this with the perceived infection risk in the population).

$$E_i(b_t, s_i, k) = b_t U_{k,i}(s_i, +) + (1 - b_t)U_{k,i}(s_i, -) \tag{5}$$

The actor chooses between US and PS according to a Fermi-Dirac equation (Equation 6) for the probability $f(\Delta E)$ of choosing US:

$$f(\Delta E) = \frac{1}{1 + e^{-(\Delta E)}} \tag{6}$$

where ΔE represents the difference between expected utilities. Actor 1 formulates ΔE as $\Delta E =$
$E_i(b_t, s_i, US)$ - $E_i(b_t, s_i, PS)$, representing the difference in expected utilities calculated for US and PS. According to the Fermi-Dirac equation, when the value of ΔE is large and positive, or large and negative, then there is a high probability the individual will choose US, or PS, respectively. However, if ΔE is small, then the actor could choose US even though $\Delta E < 0$, or PS even though $\Delta E > 0$. This provides a more realistic model of decision-making whereby if the utility difference is small, factors other than the expressed utility (such as personal preferences or other sources of "randomness") enter into decision-making. We choose this functional form because it is relatively simple, and has been used in previous models of decision-making [47, 48]. For every decision made, a random value is

created between 0 and 1. If $f(\Delta E)$ is greater than this value, the individual's choice becomes US, if it is less than this, PS is the action chosen. For Actor 2, they choose between the offer given to them by Actor 1 and NS, meaning their $\Delta E = E_i(b_t, s_i, US)$ - $E_i(b_t, s_i, NS)$ or $\Delta E = E_i(b_t, s_i, PS)$ - $E_i(b_t, s_i, NS)$.

Understanding the evolution of b_t-values

This section describes the evolution of b_t-values through gameplay. Supplementary Table S4 visualizes how the game is played. Randomly selected Actor 1 will weigh their expected utilities to offer either US or PS. Actor 2 either increases or decreases their b_t-value according to Equations 1 and 2, then chooses their option between what was offered to them or NS. After Actor 2 has made their offer, Actor 1 will either increase their b_t-value if offered US or decrease it and accept the choice. The structure of the risky sex game was meant to portray interactions between highly active individuals with strangers of a similar non-monogamous life style. Having individuals choose solely between Actor 1s offer and NS was based on the assumption that these are quick one-off interactions with limited verbal interaction. As such, the Actor 2 is limiting their response as a result of not being able to engage in a lengthy negotiation. In support of this assumption, Elwood *et al*, reported a social norm for silence in bathhouse public areas. The norm facilitates efficient and anonymous sexual encounters, but precludes the ability to negotiate condom use verbally [49].

Choice threshold according to assigned utilities (No intervention)

An HIV+ person will always prefer US to all other options, and PS when deciding between PS and NS. The HIV- individuals' choice varies depending on what their b_t-value is. According to baseline utilities, HIV- individuals with b_t-value $< 0.\bar{3}$ prefer US to PS. When choosing between US and NS, HIV- individuals with b_t-value $< 0.\bar{6}$ prefer US to NS. HIV- individuals will always prefer PS to NS under baseline utilities.

This helps explain why the b_t-values of HIV- individuals reach a ceiling in many simulations. When their values are $< 0.\bar{3}$ They prefer US and will subsequently increase their b_t-value when they are Actor 1, or Actor 2 when being offered US. However, when their b_t-value surpasses $0.\bar{3}$ They will choose PS when they are Actor 1 and subsequently decrease their b_t-value when Actor 2 inevitably accepts. Since every actor is randomly assigned to be Actor 1 or 2 at every instance of the game, their b_t-value continues to oscillate between this value. The overall average b_t-value may appear slightly higher as individuals who dip below $< 0.\bar{3}$ and choose US may contract HIV and be removed from the average resulting in a slightly higher overall average. Under the current utilities assigned, when choosing between PS and NS, no individual has incentive to choose NS, as we would expect. HIV- individuals choosing between US and NS will choose US whenever their b_t-value $< 0.\bar{6}$, according to the baseline utilities. Because of the ceiling occurring at $0.\bar{3}$ the choice for NS would only occur by either adjusting the utilities or the parameters for the Fermi-Dirac equation. For HIV+ individuals, since US is always the favoured option, increased prevalence increases their overall average b_t-value, see Figure S2.

Choice threshold according to assigned utilities (HAART)

Upon introducing HAART into the population, the U_{US} for an HIV- individual interacting with an HIV+ individual changes to Equation 3. Under the baseline settings, the threshold for an HIV- individual deciding between US and PS increases to 0.37, and the threshold between US and NS increases to 0.74. This explains why we see an increase in the overall average HIV- b_t-value threshold when compared to the no intervention option. It also explains why increasing τ, the transmission probability, increases prevalence but does not increase the average overall b_t-value beyond the established threshold as seen in Figure S9.

Choice threshold according to assigned utilities (Vaccine intervention)

The results for introducing HIV vaccine are more diverse since the duration of vaccine protection has a specified length, which may also differ from an individual's belief that they are protected by the vaccine. The utility assigned for an HIV- individual that believes they are protected changes from -50 to a value of 100, this means that they always prefer US as long as they believe they are protected, even if the protection of the vaccine has ended. A vaccinated individual checks their protection status at 5 and 10 years into the program, and stops assuming protection after 15 years. The protection is assigned through a random-normal distribution with a mean of 10 years, and standard deviation of 3 years. When offered US, the b_t-value of an HIV- individual adjusts using Equation 4, where ρ is the vaccine coverage. For this model, we assume individuals have perfect knowledge of the age of the person they are interacting with. As the mean length of the vaccine is 10 years, individuals will formulate Equation 4 with ρ as the chosen coverage level when interacting with individuals less than 25 years of age. When interacting with individuals over 25 years of age, ρ will be set to 0 within this equation. This creates a number of situations.

1. For young individuals, the b_t-value is slower to increase under US requests according to Equation 4 and our baseline settings.

2. In addition to reducing the incremental increase when offered US, the new equation creates a threshold of 0.25 for these young individuals when deciding between US and PS. An HIV- individual's b_t-value will decrease when offered US according to our equation if the individuals b_t-value is > 0.25. This fact, combined with 1) explains why the average b_t-value is lower for the youngest age groups, (see Figure 5).

3. However, as an individual ages and begins to interact with individuals older than 25, the ρ value in Equation 4 drops to 0. Leaving HIV- individuals with a preference for US and a sharply increasing b_t-value. What is interesting is that we also encounter individuals who still believe they are protected even though their vaccine protection has expired and they are between check-ups. This creates a group with a preference for US despite not having protection, even though they assume individuals over 25 to no longer be protected. This rapidly increases their b_t-value as these individuals are always

preferring US and continue to increase their b_t-value when interacting with people over 25 years of age.

4. As HIV- individuals continue to age, their b_t-value continues to rise which is why we see a spike in the average b_t-value for 30-40 year-olds. All vaccinated individuals have a final check-up after 15 years from vaccination where an HIV- individual would then revert back to their original utility settings. Through interactions, their average b_t-value eventually returns to the threshold we have seen in the no intervention option as seen in the older age groups (see Figure 5).

Interesting points

We make the following observations about the evolution of the b_t-values.

1. The threshold found in the no intervention and HAART option is arguably a realistic portrayal of how an HIV- individual who randomly alternates between proposing and being propositioned would view their risk-assessment. An increase in threshold through HAART is also realistic since HAART aims to reduce the risk of spread, allowing HIV- individuals for an increased adjustment in their utility.

2. For the vaccination program, it is very interesting to see the effects of a population that understands the limitations of the length of the vaccine (assumes anyone older than 25 is no longer protected), but may overestimate their coverage between check-ups. The increase in average b_t-value for HIV- 30-40 year-olds reflects the consequences of individuals that prefers US, believing they are vaccinated while conservatively assuming the vaccination of others ended after 10 years. 30-40 year-olds having an increased b_t-value and thus preferring PS is beneficial as this results in less chances to contract HIV or other sexually transmitted infections.

3. A booster program or catch-up vaccination scenario would likely shift the average increased b_t-value average from 30-40 year-old's to a higher age group. Since these age groups are still sexually active, it would likely be beneficial to implement such a program.

Age	Prob. of overall activity
15-19	$\delta = 0.5$
20-29	$\delta = 1$
30-39	$\delta = 0.8$
40-49	$\delta = 0.6$
50-59	$\delta = 0.3$
60-69	$\delta = 0.1$
70-79	$\delta = 0$

Table S1: Multiplicative factor δ controlling overall sexual activity level relative to 20-29-year-olds, by age.

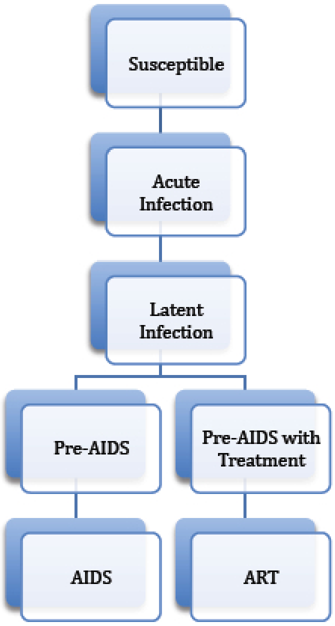

Figure S1: Flow diagram outlining how individuals progress to AIDS once infected, both with and without treatment.

Stage	Definition	Value	Source
ω	age of the individual. This stage ends after 60 years or age 79	15-79 years	[34]
ω_{Acu}	duration of time in acute stage. Once infected an individual will begin to age in the acute stage while exhibiting γ_{Acu} relative influence of transmission. This will last for 5 months unless their age reaches 79.	5 months	[34]
ω_{Lat}	duration of time in latent stage. After Acute stage an individual will begin to age in the latent stage while exhibiting γ_{Lat} relative influence of transmission. This will last for 90 months unless total age reaches 79.	90 months	[34]
ω_{P-AIDS}	duration of time in pre-AIDS stage. After Latent stage an individual will begin to age in the pre-aids stage while exhibiting γ_{P-AIDS} relative influence of transmission. This will last for 24 months unless total age reaches 79.	24 months	[34]
ω_{AIDS}	duration of time in AIDS stage. After pre-AIDS an individual will begin to age in the AIDS stage while exhibiting γ_{AIDS} relative influence of transmission. This will last for 60 months unless total age reaches 79. If individual reaches end of this stage, it will die.	60 months	[34]
$\omega_{P-AIDS-H}$	duration of time in pre-AIDS stage, with HAART. After Latent stage, if individual is selected for treatment the individual will begin in the pre-AIDS with treatment stage while exhibiting $\gamma_{P-AIDS-H}$ relative influence of transmission. This will last for 2 months unless total age reaches 79.	2 months	[34]
ω_{AIDS-H}	duration of time in AIDS stage, with HAART. After pre-AIDS with treatment stage an individual will begin in the AIDS with treatment stage while exhibiting γ_{AIDS-H} level of infectiveness. This will last for 288 months unless total age reaches 79. If individual reaches end of this stage, it will die.	288 months	[50]

Table S2: Duration of HIV infection stages with and without HAART.

Term	Definition	Relative change in transmission	Source
γ_{Acu}	Relative change in transmission probability from an HIV+ actor to an HIV- actor through US during the acute stage.	11	[34]
γ_{Lat}	Relative change in transmission probability from an HIV+ actor to an HIV- actor through US during the latent stage.	1	[34]
γ_{P-AIDS}	Relative change in transmission probability from an HIV+ actor to an HIV- actor through US during the pre-AIDS stage.	3.3	[34]
γ_{AIDS}	Relative change in transmission probability from an HIV+ actor to an HIV- actor through US during the AIDS stage.	6.1	[34]
$\gamma_{P-AIDS-H}$	Relative change in transmission probability from an HIV+ actor to an HIV- actor through US during the pre-AIDS with HAART stage.	0.5	[51]
γ_{AIDS-H}	Relative change in transmission probability from an HIV+ actor to an HIV- actor through US during the AIDS with HAART stage.	0.1	[34]

Table S3: Relative reduction in transmission probability depending on stage of infection. The baseline HIV transmission probability τ (see Table 2) is modified by these values to produce the actual stage-specific HIV transmission rate.

Actor	Action				
Actor 1	First offer	US		PS	
Actor 2	b_t update	$\uparrow$ b		$\downarrow$ b	
	Second offer	US	NS	PS	NS
Actor 1	b_t update	$\uparrow$ b	$\downarrow$ b	$\downarrow$ b	$\downarrow$ b
	Result	US	NS	PS	NS

Table S4: Layout of risky sex game between randomly selected actors. Actor 1 makes the first offer between US and PS. Actor 2 updates their b_t-value using equation 1 or 2. Actor 2 then makes an offer consisting of either Actor 1's offer or NS. Actor 1 then updates their b_t-value and the resulting action occurs.

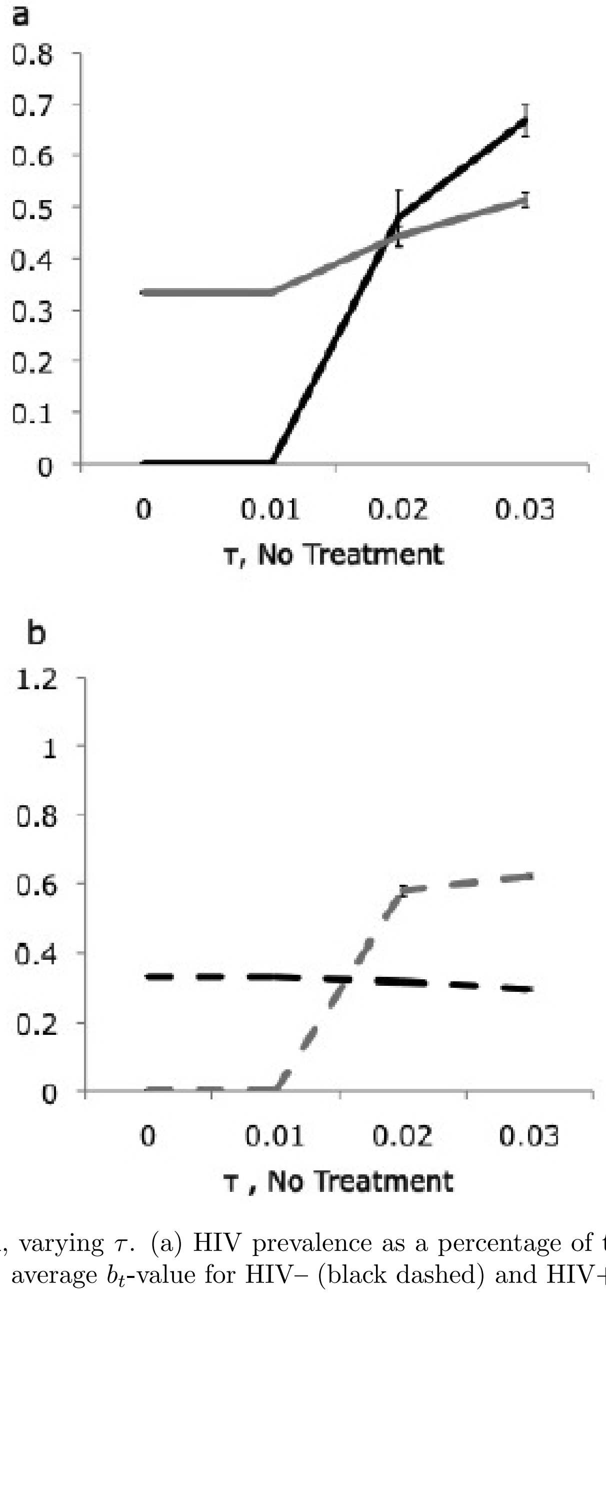

Figure S2: No intervention, varying τ. (a) HIV prevalence as a percentage of the population (black), total average b_t-value (grey); (b) average b_t-value for HIV− (black dashed) and HIV+ (grey dashed) populations.

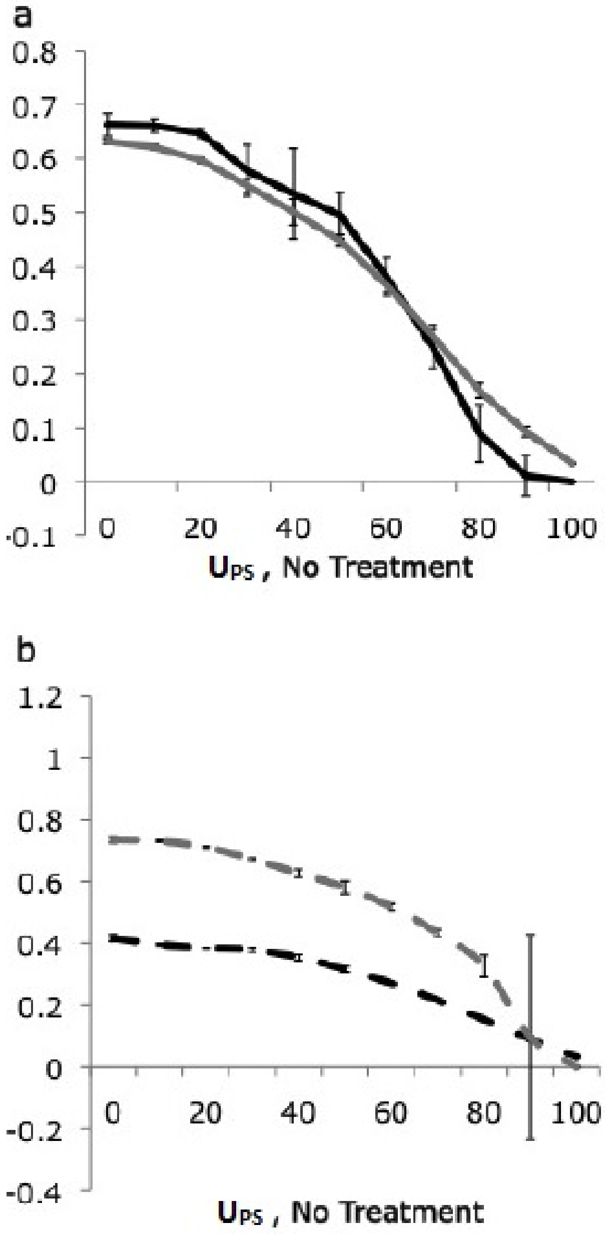

Figure S3: No intervention, varying U_{PS}, (a) HIV prevalence as a percentage of the population (black), total average b_t-value (grey); (b) average b_t-value for HIV− (black dashed) and HIV+ (grey dashed) populations.

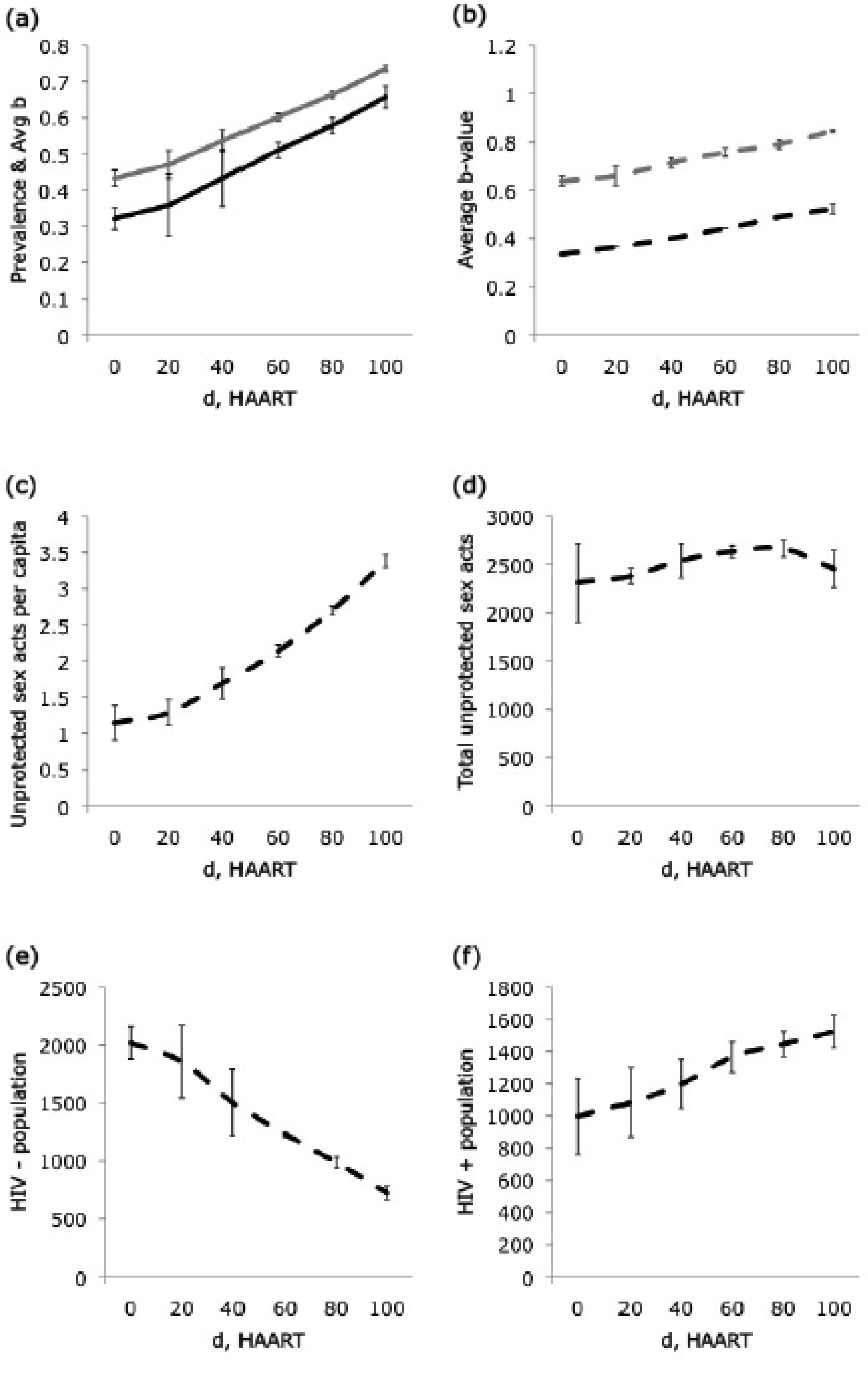

Figure S4: HAART intervention, varying d. (a) HIV prevalence as a percentage of the population (black), total average b_t-value (grey); (b) average b_t-value for HIV− (black dashed) and HIV+ (grey dashed) populations. (c) total number of -/+ US acts (number of unprotected sex acts between HIV- and HIV+ pairs) per year divided by number of HIV− individuals at the end of the year; (d) total number of -/+ US acts per year; (e) number of HIV− individuals; (f) number of HIV+ individuals at end of simulation.

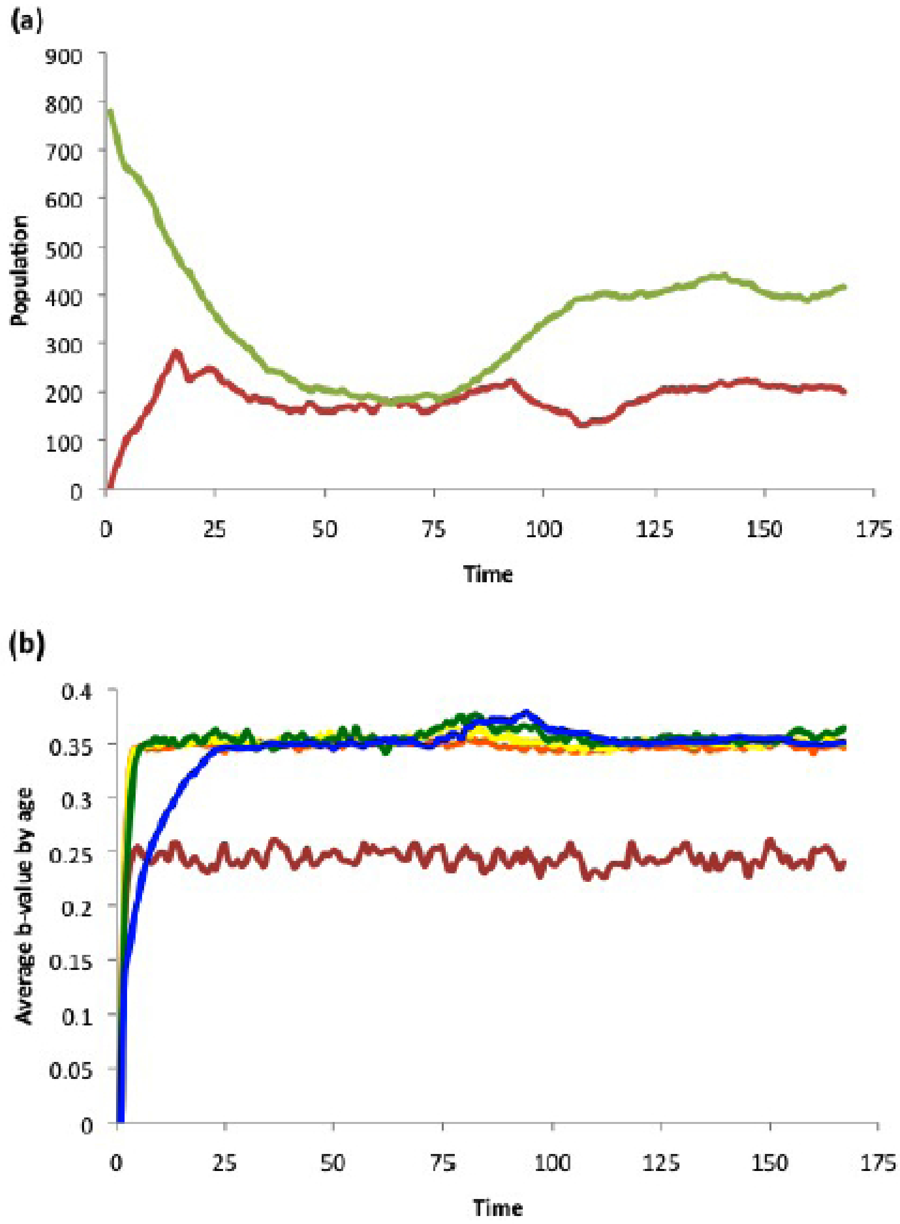

Figure S5: HAART intervention, d=0. (a) number of HIV− (green) and HIV+ (red) individuals in the population; (b) average b_t-value for HIV− individuals by age, 15-20-year-olds (red), 20-30 (orange), 30-40 (yellow), 40-50 (green), and 50+ year-olds (blue).

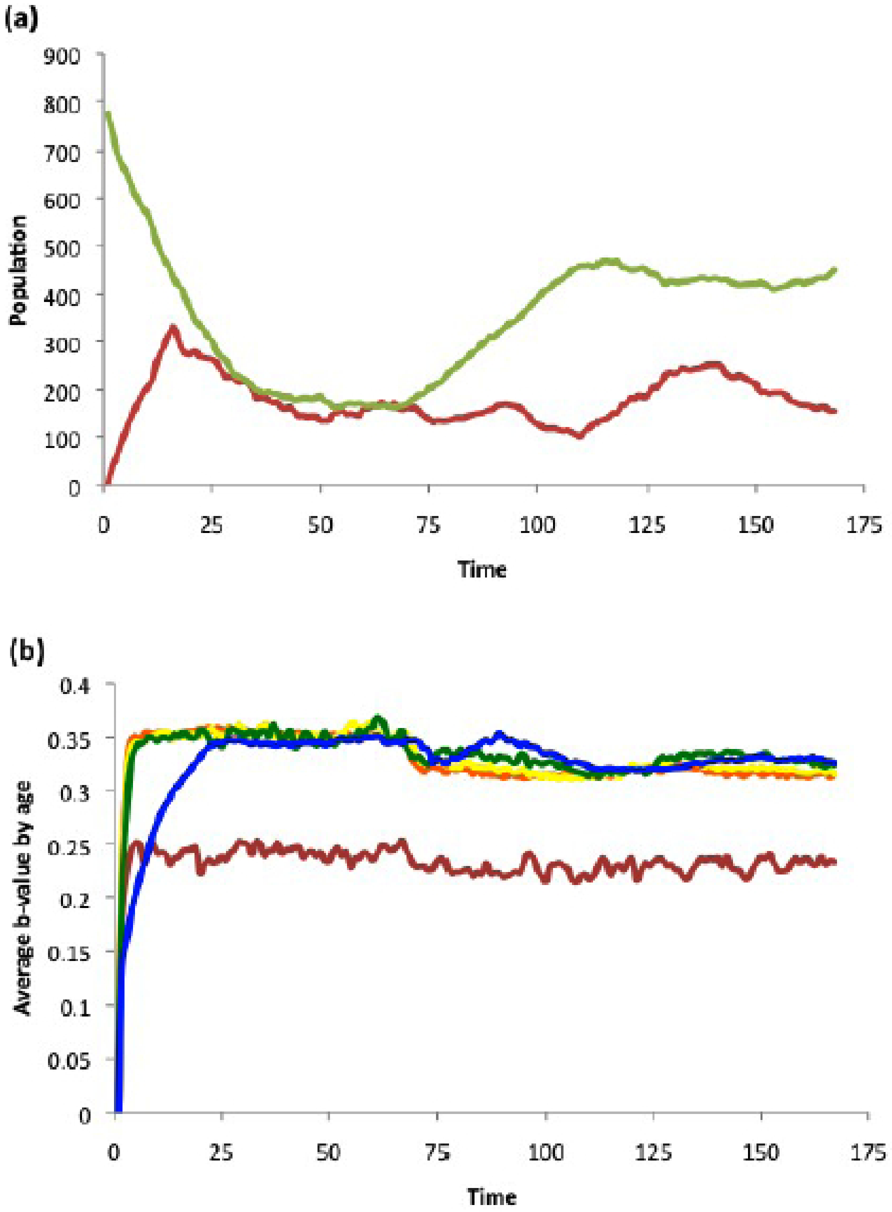

Figure S6: HAART intervention, d=-20. (a) number of HIV− (green) and HIV+ (red) individuals in the population; (b) average b_t-value for HIV− individuals by age, 15-20-year-olds (red), 20-30 (orange), 30-40 (yellow), 40-50 (green), and 50+ year-olds (blue).

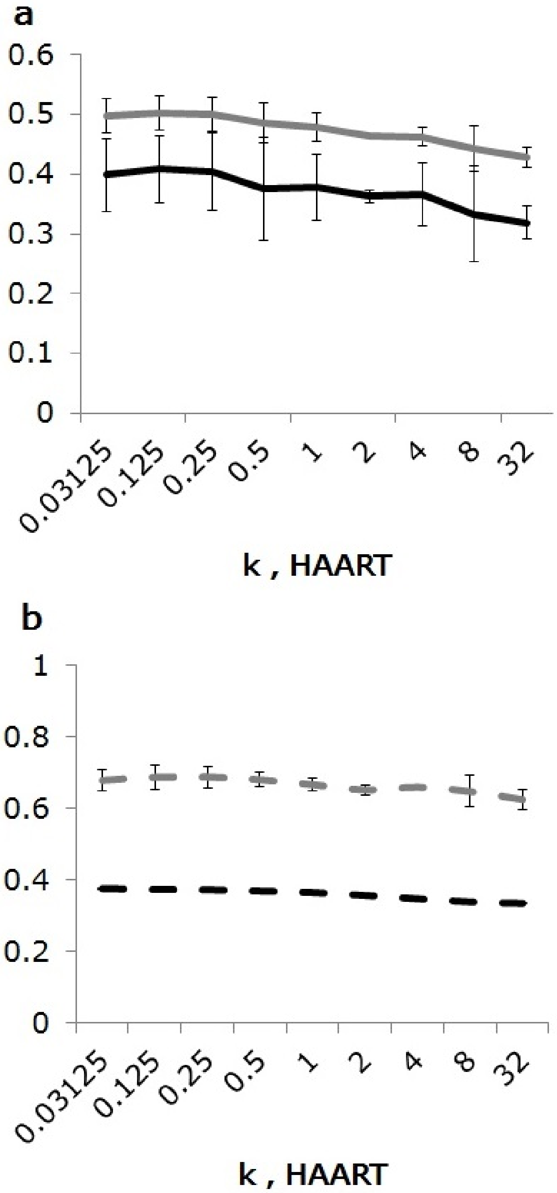

Figure S7: HAART intervention varying k. (a) HIV prevalence as a percentage of the population (black), total average b_t-value (grey); (b) average b_t-value for HIV– (black dashed) and HIV+ (grey dashed) populations.

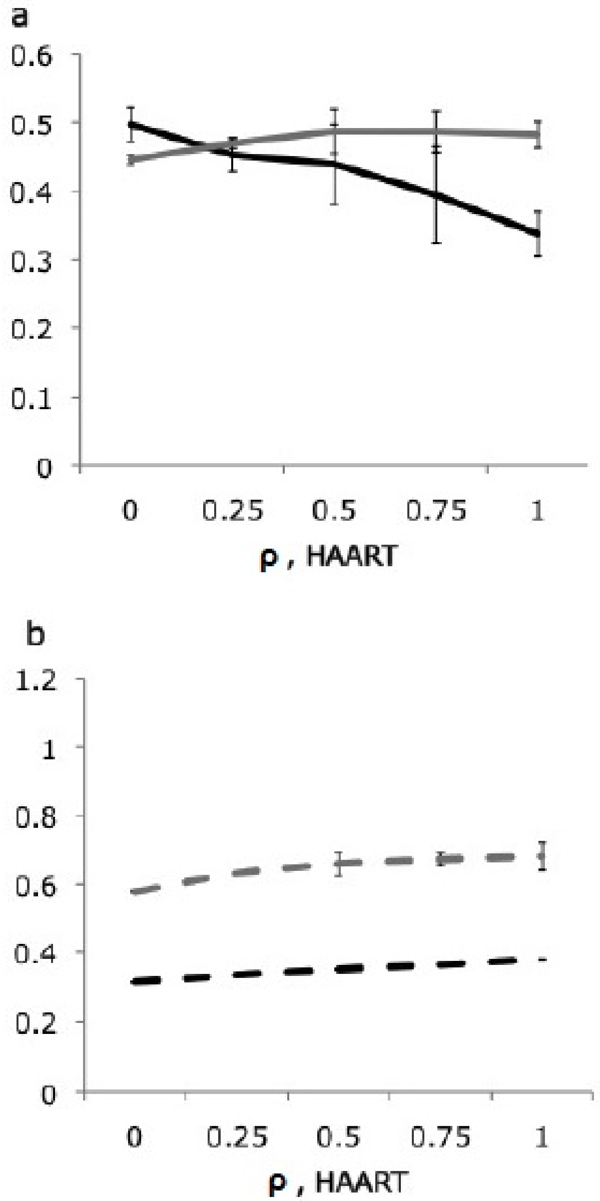

Figure S8: HAART varying ρ. (a) HIV prevalence as a percentage of the population (black), total average b_t-value (grey); (b) average b_t-value for HIV− (black dashed) and HIV+ (grey dashed) populations.

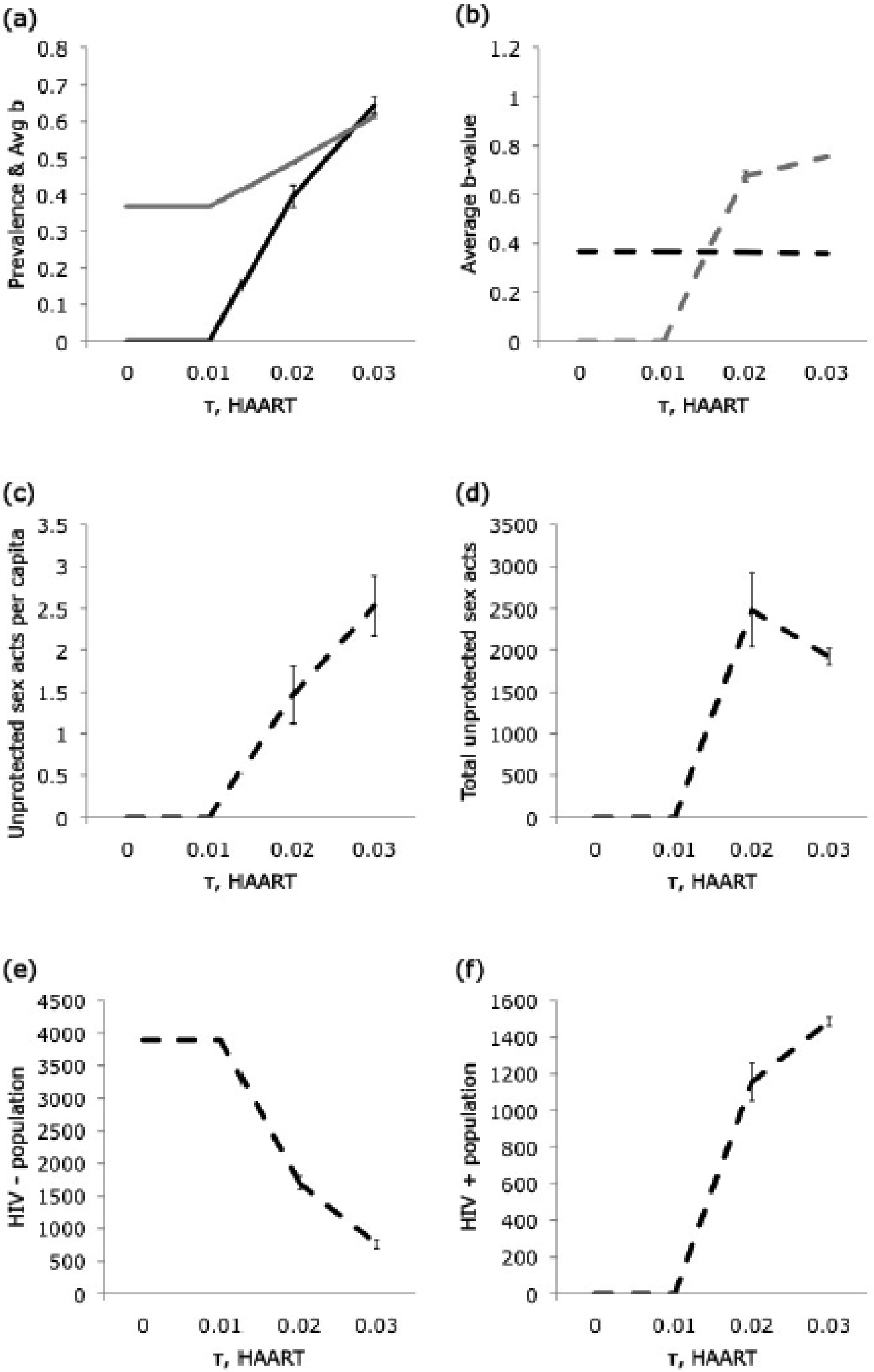

Figure S9: HAART intervention, varying τ. (a) HIV prevalence as a percentage of the population (black), total average b_t-value (grey); (b) average b_t-value for HIV− (black dashed) and HIV+ (grey dashed) populations. (c) total number of -/+ US acts (number of unprotected sex acts between HIV- and HIV+ pairs) per year divided by number of HIV− individuals at the end of the year; (d) total number of -/+ US acts per year; (e) number of HIV− individuals; (f) number of HIV+ individuals at end of simulation.

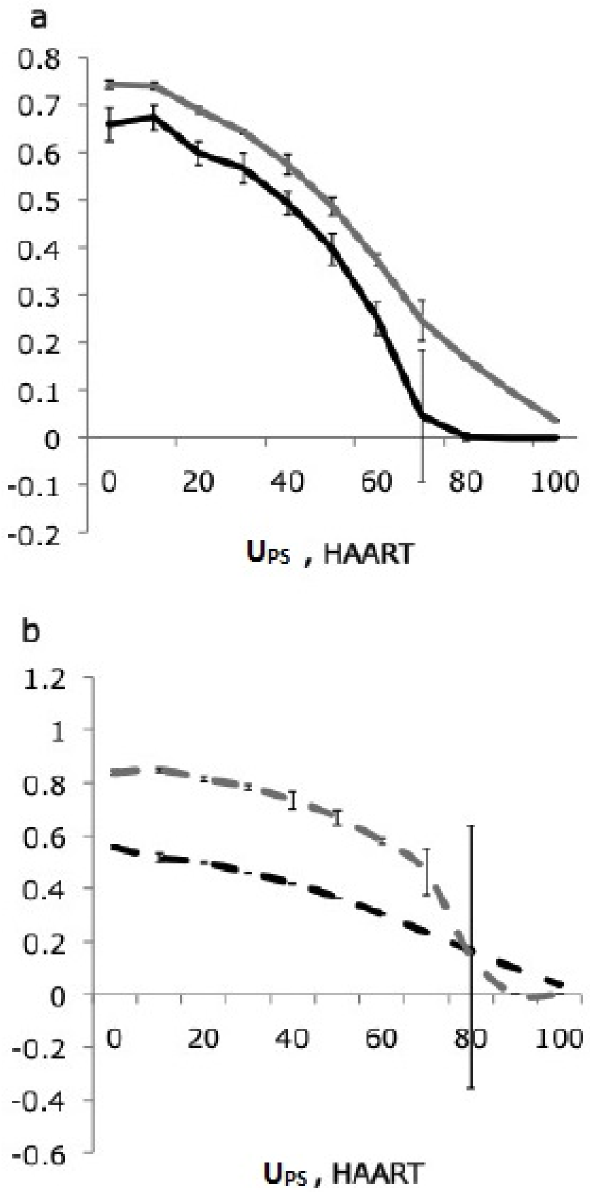

Figure S10: HAART intervention, varying U_{PS}, (a) HIV prevalence as a percentage of the population (black), total average b_t-value (grey); (b) average b_t-value for HIV− (black dashed) and HIV+ (grey dashed) populations.

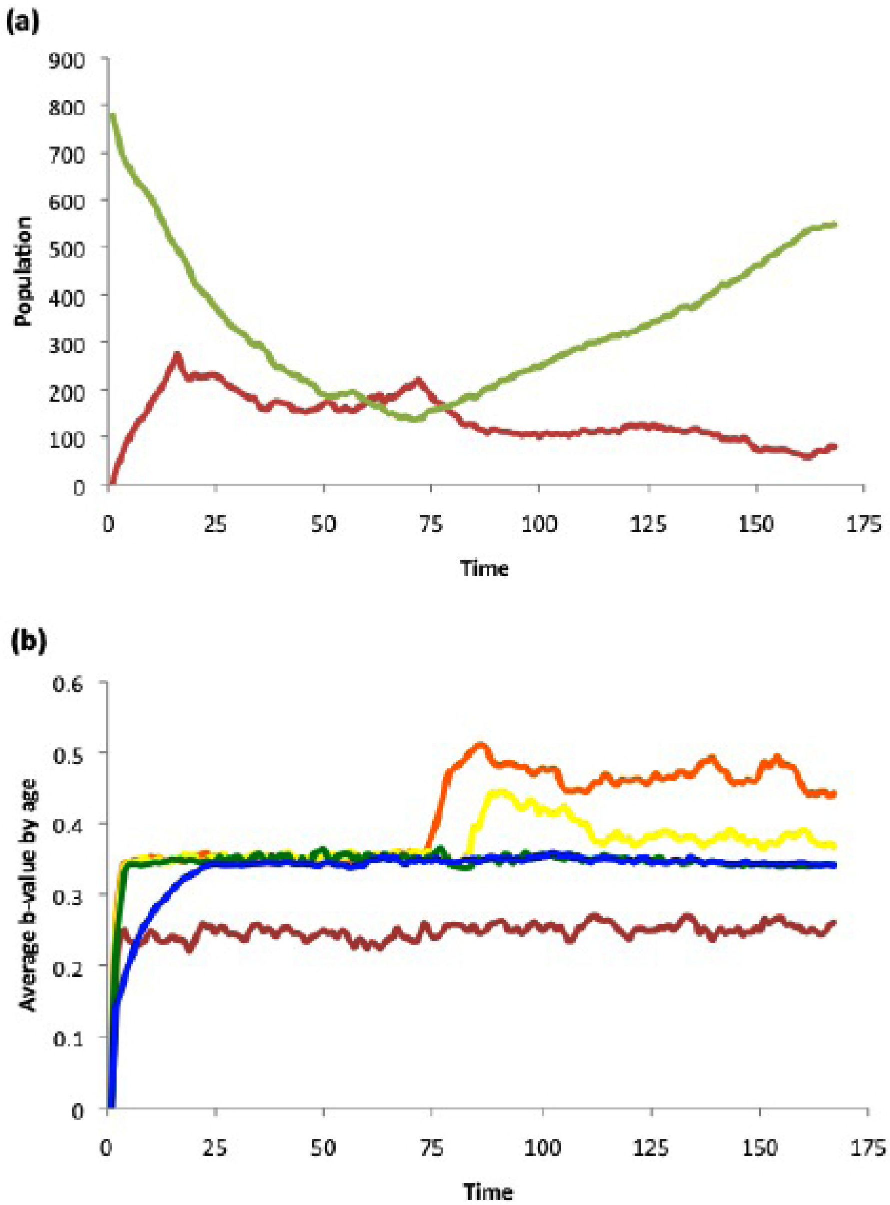

Figure S11: Vaccination Intervention, k=8. (a) number of HIV− (green) and HIV+ (red) individuals in the population; (b) average b_t-value for HIV− individuals by age, 15-20-year-olds (red), 20-30 (orange), 30-40 (yellow), 40-50 (green), and 50+ year-olds (blue).

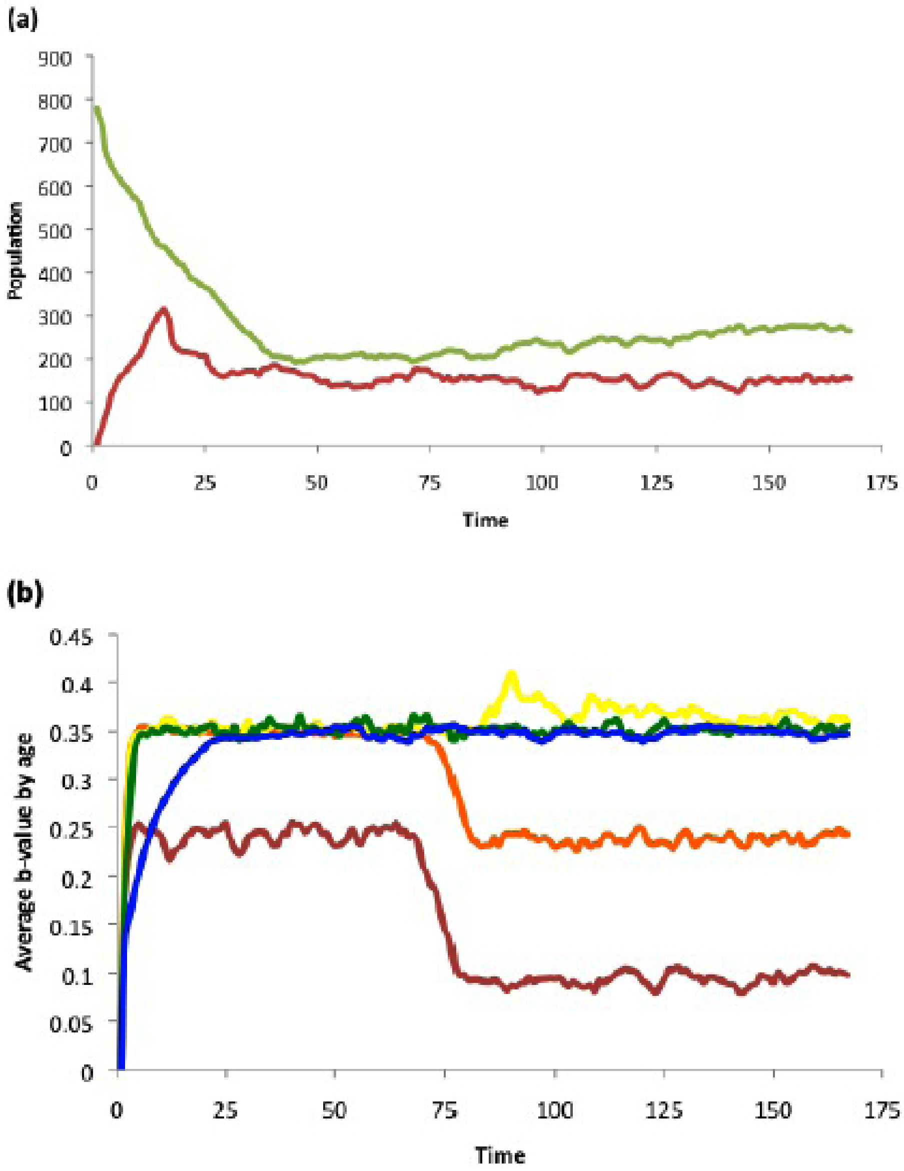

Figure S12: Vaccination Intervention, k=0.125. (a) number of HIV− (green) and HIV+ (red) individuals in the population; (b) average b_t-value for HIV− individuals by age, 15-20-year-olds (red), 20-30 (orange), 30-40 (yellow), 40-50 (green), and 50+ year-olds (blue).

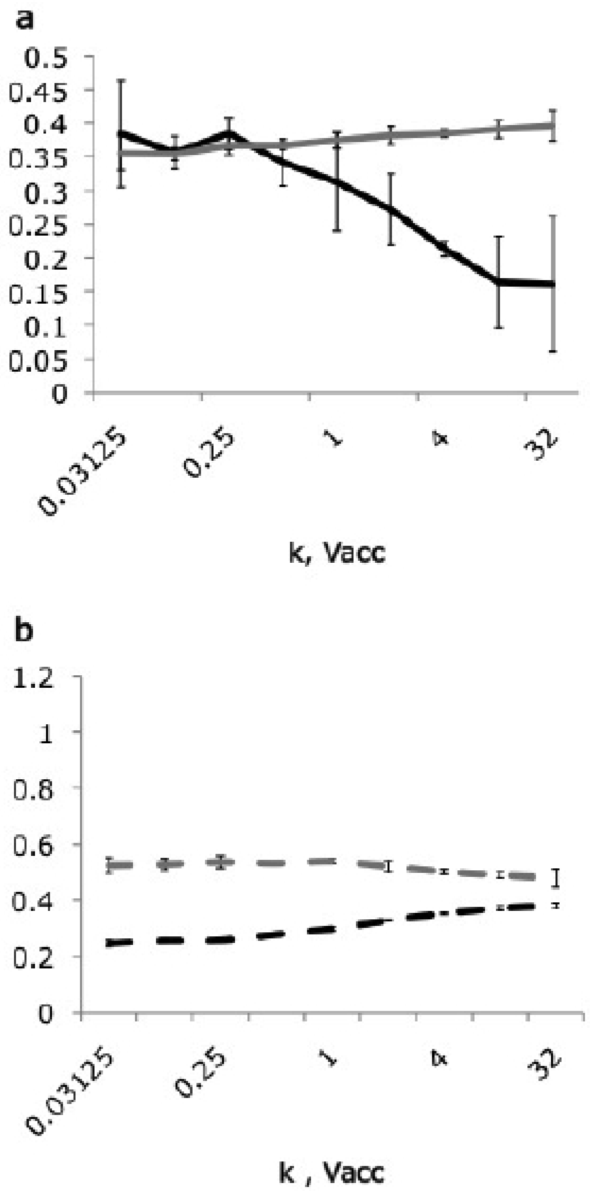

Figure S13: Vaccine intervention, varying k, (a) HIV prevalence as a percentage of the population (black), total average b_t-value (grey); (b) average b_t-value for HIV− (black dashed) and HIV+ (grey dashed) populations.

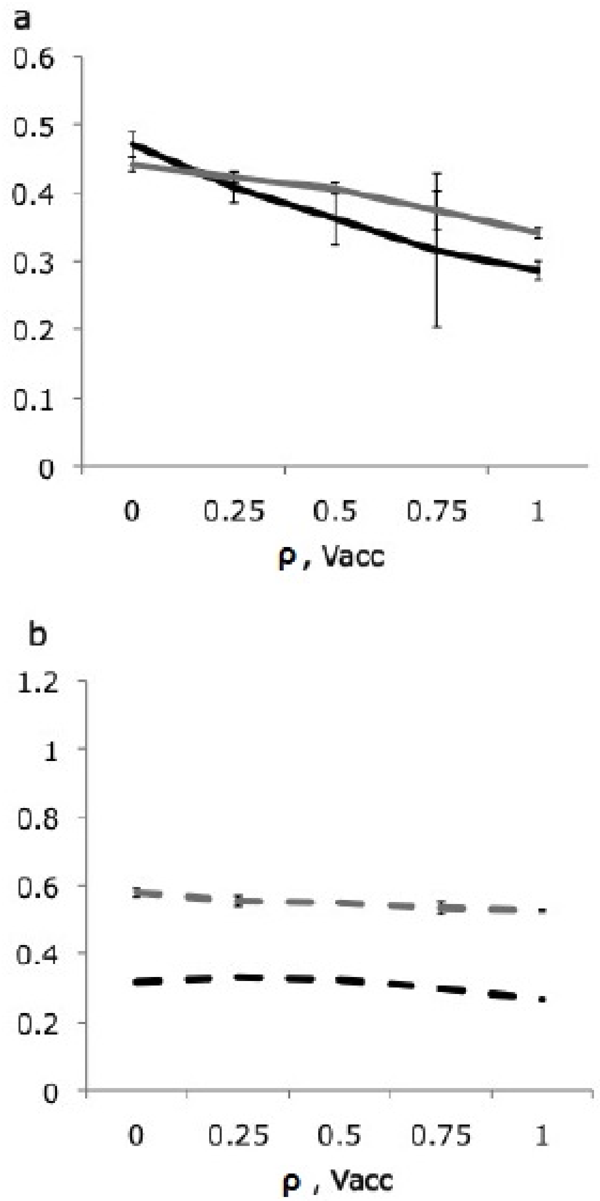

Figure S14: Vaccine intervention, varying ρ, (a) HIV prevalence as a percentage of the population (black), total average b_t-value (grey); (b) average b_t-value for HIV− (black dashed) and HIV+ (grey dashed) populations.

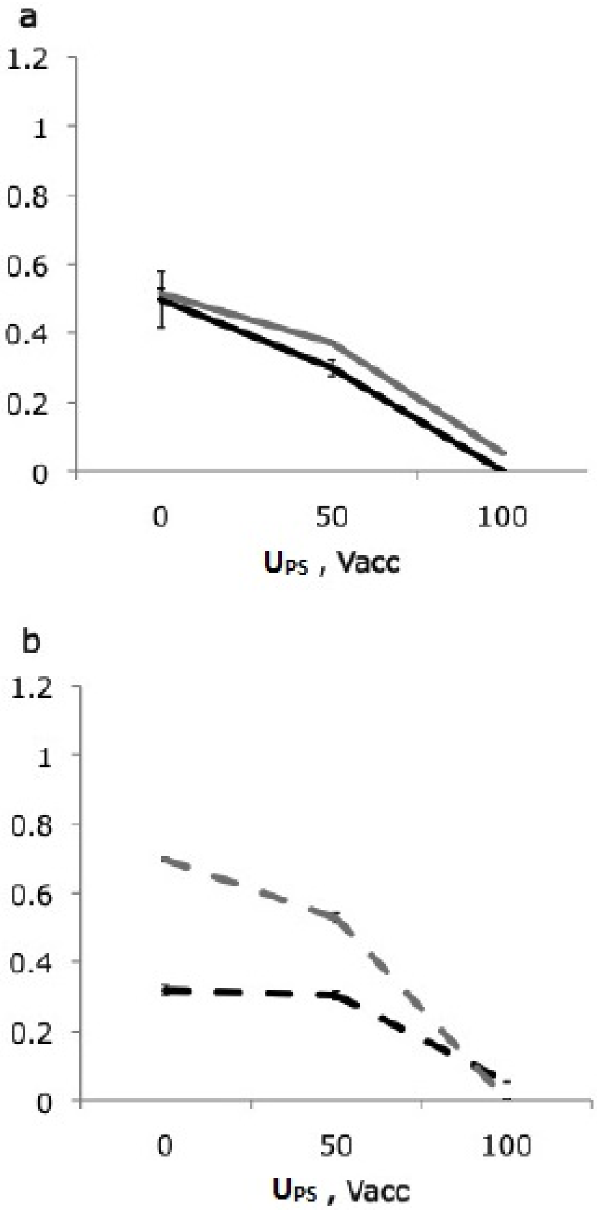

Figure S15: Vaccine intervention, varying U_{PS}, (a) HIV prevalence as a percentage of the population (black), total average b_t-value (grey); (b) average b_t-value for HIV− (black dashed) and HIV+ (grey dashed) populations.

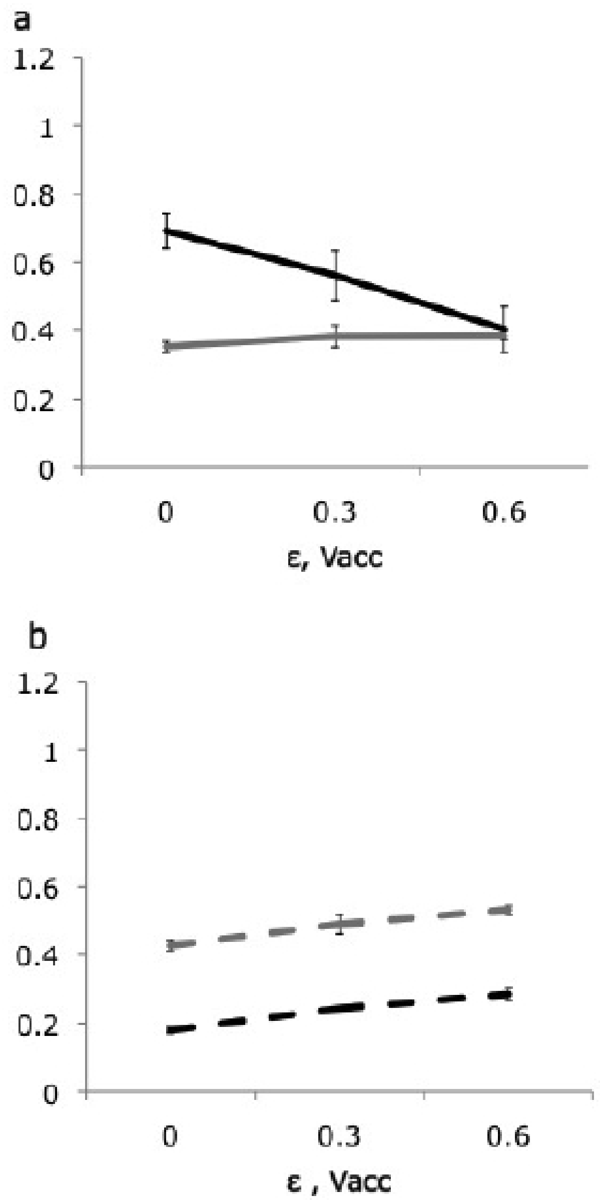

Figure S16: Vaccine intervention, varying ϵ, (a) HIV prevalence as a percentage of the population (black), total average b_t-value (grey); (b) average b_t-value for HIV− (black dashed) and HIV+ (grey dashed) populations.

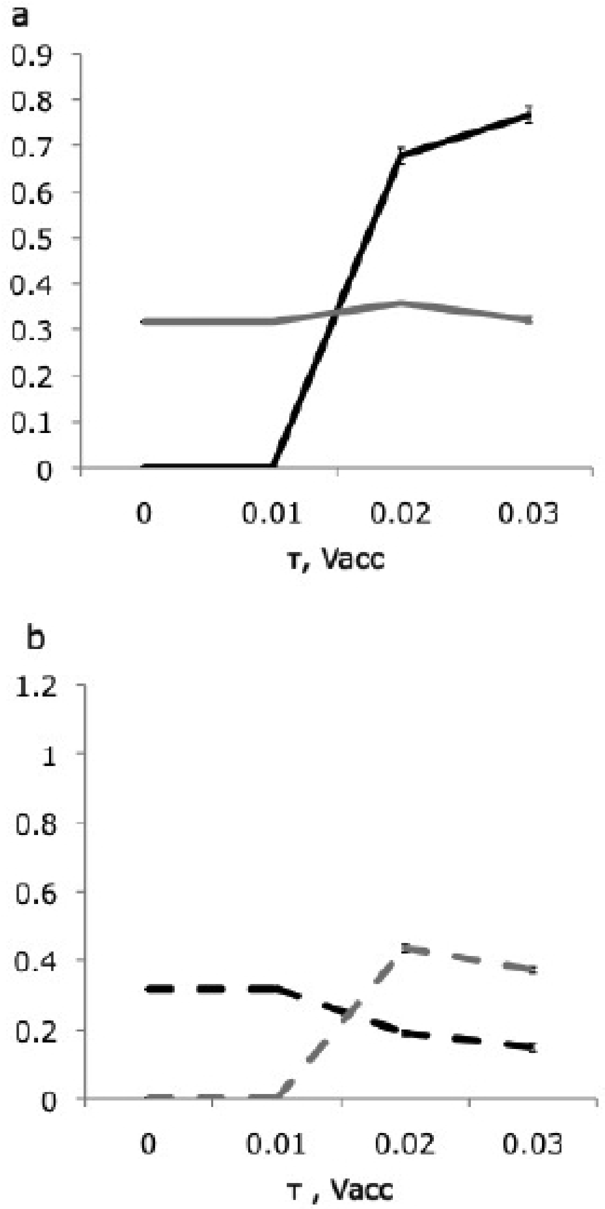

Figure S17: Vaccine intervention, varying τ, (a) HIV prevalence as a percentage of the population (black), total average b_t-value (grey); (b) average b_t-value for HIV− (black dashed) and HIV+ (grey dashed) populations.
